# Nature-inspired IL-1 targeted therapy to treat chronic inflammatory diseases

**DOI:** 10.1016/j.ymthe.2025.09.008

**Published:** 2025-09-10

**Authors:** Yeon-Suk Yang, Mi-Jeong Kim, Sachin Chaugule, Emma Mayer, Ngoc DeSouza, Hong Ma, Jun Xie, Ki-Young Lee, Shaoguang Li, Ellen Gravallese, Guangping Gao, Jae-Hyuck Shim

**Affiliations:** 1 Department of Medicine, UMass Chan Medical School, Worcester, MA, USA; 2 Department of Genetic and Cellular Medicine, UMass Chan Medical School, Worcester, MA, USA; 3 Horae Gene Therapy Center, Worcester, MA, USA; 4 Viral Vector Core, UMass Chan Medical School, Worcester, MA, USA; 5 Department of Microbiology and Physiological Systems, UMass Chan Medical School, Worcester, MA, USA; 6 Department of Immunology and Samsung Biomedical Research Institute, Sungkyunkwan University School of Medicine, Suwon, Korea; 7 Division of Rheumatology, Inflammation, and Immunity, Department of Medicine, Brigham and Women’s Hospital, Boston, MA 02115, USA; 8 Li Weibo Institute for Rare Diseases Research, UMass Chan Medical School, Worcester, MA, USA

**Keywords:** rheumatoid arthritis, autoinflammatory diseases, AAV, sIL-1Ra, DIRA, bone loss, articular bone erosion

## Abstract

The interleukin (IL)-1 pathway is a key mediator of inflammation and innate immune responses. Its dysregulation contributes to rheumatoid arthritis (RA) and autoinflammatory diseases (AIDs). In this study, we develop a recombinant adeno-associated virus (rAAV)-based gene therapy to deliver an inflammation-inducible, secreted human IL-1 receptor antagonist (sIL-1Ra) as a complementary approach to existing IL-1 blockers. rAAV-mediated expression of sIL-1Ra dampens IL-1 signaling and inflammatory arthritis in mice. As the expression of endogenous sIL-1Ra is tightly regulated by inflammation, we developed an rAAV vector that produces sIL-1Ra in response to pro-inflammatory cytokines and bone morphogenic proteins (BMPs) enriched in the inflamed joints of patients with RA. Remarkably, inflammation-inducible sIL-1Ra is more effective than constitutively expressed sIL-1Ra in ameliorating inflammatory arthritis in the mouse model of RA. These mice showed a significant reduction in circulating immune cells, expression of the genes associated with inflammatory responses, joint swelling, and bone destruction. Similar to patients with deficiency of IL-1Ra (DIRA), IL-1Ra-deficient mice spontaneously develop inflammatory arthritis and skeletal abnormalities, which are almost completely reversed by a single systemic administration of the sIL-1Ra-expressing rAAV vector. Collectively, our results highlight inflammation-inducible IL-1-targeted therapy using an rAAV vector as a long-lasting, pathophysiologic treatment for chronic inflammatory diseases.

## Graphical abstract

**Figure:**
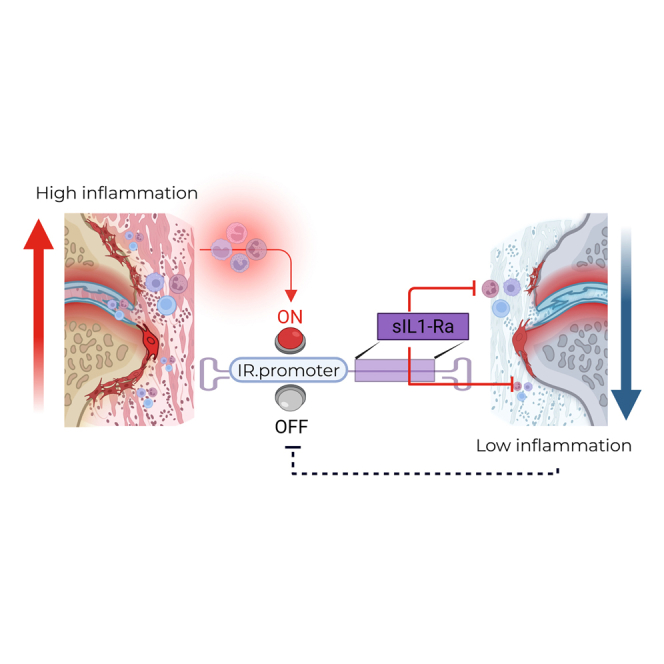

## Teaser

Treating chronic autoimmune diseases, such as rheumatoid arthritis and autoinflammatory diseases, remains challenging for drug development due to potential side effects and the requirement for long-term treatment. Shim and colleagues demonstrate that inflammation-inducible secretion of human IL-1 receptor antagonist via rAAV is a promising approach to treat inflammatory arthritis.

## Introduction

Rheumatoid arthritis (RA) is a chronic autoimmune disorder that causes severe inflammation of the joints and synovial tissues, leading to joint pain, swelling, and stiffness. Globally, RA affects approximately 18 million people and 1.3 million Americans older than age 50 live with the disease.^1^ In RA, immune cells (i.e., neutrophils, macrophages, lymphocytes, and dendritic cells) infiltrate into the synovial tissue of inflamed joints, triggering synoviocyte proliferation and the production of pro-inflammatory cytokines, followed by cartilage damage and articular bone erosions.^2^ Research into pro-inflammatory cytokines and their signaling pathways made significant advancements in the development of disease-modifying antirheumatic drugs (DMARDs) targeting tumor necrosis factor (TNF), interleukin (IL)-1, IL-6, IL-17, Janus kinases (JAKs), and T and B lymphocytes.^3^ The currently available biologic DMARDs are considered a major breakthrough in its treatment, as early and aggressive intervention can prevent joint erosion and systemic bone loss. These biologics target key immune pathways, including TNF (golimumab, certolizumab, etanercept, adalimumab, and infliximab), IL-6 (tocilizumab), T cells (abatacept), B cells (rituximab), and JAK kinases (tofacitinib).^4^^,^^5^^,^^6^^,^^7^^,^^8^ However, many patients are unable to access these agents early enough to prevent the progression of RA due to limited access to rheumatologists. Additionally, RA is a chronic inflammatory disease requiring long-term treatment, and so there is a high burden to deliver lifelong treatment to people who are prone to developing disease-induced disabilities. Thus, it is still an unmet need to develop drugs that provide long-lasting therapeutic effects with a single administration, addressing both accessibility challenges and patient burden.

Similar to RA, autoinflammatory diseases (AIDs) trigger excessive systemic inflammation, including fever, rashes, fatigue, arthritis, and multiple organ inflammation.^9^ While RA is a prevalent polygenic disease primarily caused by a malfunction of the adaptive immune system, AIDs are rare monogenic conditions caused by pathogenic mutations in key regulators of the innate immune system.^9^^,^^10^ AIDs arise from aberrant activation of pro-inflammatory cytokine pathways, including ILs, interferons (IFNs), and TNF.^11^ In particular, the hyperactivation of IL-1 signaling caused by autosomal-dominant mutations in the NLR family pyrin domain containing 3 (*NLRP3*)^12^ and autosomal-recessive mutations in *IL-1RN*^13^ result in cryopyrin-associated periodic syndromes (CAPSs) and deficiency of IL-1 receptor antagonist (DIRA),^14^ respectively. Notably, CAPS range from mild disorders such as familial cold autoinflammatory syndrome^15^ and Muckle-Wells syndrome^16^ to severe neonatal-onset multisystem inflammatory disease (NOMID).^17^ Identification of these causative mutations marked a significant advance in our understanding of the contribution of the IL-1 pathway to the pathogenesis of inflammatory diseases.^11^

The IL-1 family of cytokines is classified into three subfamilies, IL-1, IL-18, and IL-36, based on their shared interactions with receptors and co-receptors.^18^ The IL-1 subfamily members IL-1α,^19^ IL-1β,^20^ and IL-1 receptor antagonist (IL-1Ra)^21^^,^^22^ are produced by monocytes, macrophages, neutrophils, lymphocytes, and synovial fibroblasts to induce inflammation and fever while promoting synovial fibroblast proliferation and osteoclast differentiation.^23^ The expression, release, and activation of IL-1α and IL-1β are tightly regulated at multiple levels. Various damage-associated molecular patterns and pathogen-associated molecular patterns trigger the formation of an intracellular molecular sensor known as inflammasome composed of pyrin, NLRP1, NLRC4, and NLRP3.^24^ In turn, the inflammasome activates caspase proteases 1, 4/5, and 8, which cleave pro-IL-1α and pro-IL-1β.^25^ Active IL-1α and IL-1β bind to the receptor IL-1R1 and the co-receptor IL-1RAcP to transduce activation signals to nuclear factor κBs (NF-κBs) and mitogen-activated protein kinases (MAPKs), resulting in the expression of inflammatory factors, such as IL-1rn, IL-6, IL-8, Mcp-1, and Cox-2.^26^ Additionally, IL-1 signaling induces various forms of cell death,^27^ including gasdermin E-mediated cell lysis,^28^ gasdermin D-mediated pyroptosis, and mixed lineage kinase domain-like protein-mediated necroptosis.^29^ In contrast, IL-1Ra, a naturally occurring inhibitor of IL-1, is upregulated in response to inflammatory stimuli to act as a negative feedback regulator by inhibiting IL-1α and IL-1β from binding to IL-1R1.^30^ Of note, injection of recombinant IL-1β into healthy rabbit joints induces inflammatory arthritis^31^ and anti-IL-1β antibody treatment ameliorates collagen-induced arthritis (CIA) in mice.^32^ Additionally, IL-1Ra-deficient mice spontaneously develop inflammatory arthritis with synovial inflammation, articular bone erosions, and elevated levels of proinflammatory cytokines (i.e., TNF, IL-6, and IL-1β).^33^ In line with this, recombinant IL-1Ra (anakinra) has been used to treat RA patients aged 18 years or older who have failed one or more DMARDs^18^ as well as for patients with CAPS and DIRA.^34^

Recombinant adeno-associated virus (rAAV) has been developed as an *in vivo* gene therapy vector with high-efficiency transduction of multiple organs, long-term durability of therapeutic gene expression, low post-infection immunogenicity, and good safety profiles in clinical studies.^35^ Here, we demonstrate rAAV-mediated suppression of IL-1 signaling as a viable alternative to traditional drugs for treating chronic inflammatory diseases. With a single systemic administration, inflammation-inducible, secreted human IL-1Ra effectively inhibits IL-1 signaling, limits systemic and joint inflammation, reduces systemic bone loss and articular bone erosions, and ameliorates skeletal abnormalities in mouse models of RA and DIRA. Unlike short-acting recombinant secreted human IL-1 receptor antagonist (sIL-1Ra; anakinra) therapy, which requires daily subcutaneous injections, a single treatment of our AAV gene therapy provides long-lasting, inflammation-inducible expression of sIL-1Ra. This approach minimizes treatment-related adverse effects while offering sustained, nature-like, therapeutic benefits for patients with RA and/or DIRA.

## Results

### Development of an inflammation-inducible rAAV vector

**Figure 1:**
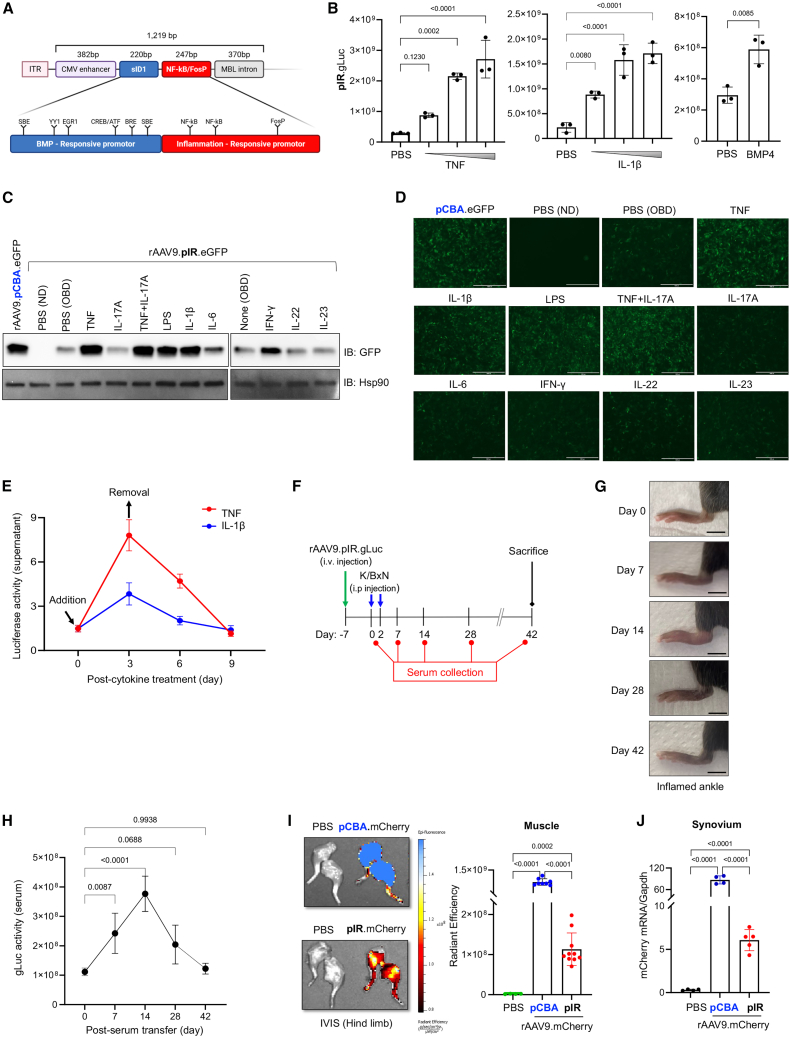
Development of AAV vector with inflammation-inducible expression (A) Schematic diagram showing the inflammation-inducible promoter (pIR). The pIR construct consists of a 382-bp CMV enhancer, a 220-bp short *Id1* promoter, a 247-bp *PB2* promoter, and a 370-bp MBL intron. The pIR sequence includes two SMAD-binding elements (SBEs), one BMP-responsive element (BRE), and the binding sites for one YY1, one EGR1, one CREB/ATF, two NF-κB, and one FosP. CMV, cytomegalovirus; ID1, inhibitor of differentiation/DNA binding 1; ITR, inverted terminal repeat. (B) *In vitro* validation of the pIR-gLuc reporter plasmid. HEK293 cells were transiently transfected with the pIR-gLuc plasmid and stimulated with varying concentrations of human TNF or IL-1β or human BMP4, and a luciferase assay was performed to measure gLuc activity (*n* = 3). PBS was used as a negative control. (C and D) Primary calvarial osteoblasts (COBs) were transduced with rAAV9 carrying pCBA.EGFP or pIR.EGFP and cultured under non-differentiating (ND) or osteogenic (OBD) conditions in the presence or absence of various inflammatory cytokines. Two days later, GFP expression was assessed via immunoblotting using an anti-GFP antibody. Anti-HSP90 was used as a loading control (C). Alternatively, GFP expression was visualized by fluorescence microscopy (D). Scale bar: 100 μm. PBS was used as a negative control. (E) pIR.gLuc-treated COBs were incubated with human TNF or IL-1β for 3 days and switched with fresh medium. Supernatants were collected every 3 days for luciferase assay (*n* = 3). (F–H) Diagram of the study and treatment methods. Two-month-old wild-type (WT) mice were injected i.p. with arthritic K/BxN serum at days 0 and 2 after i.v. injection of rAAV9.pIR.gLuc (day −7) and euthanized at day 42. Cheek bleeding was performed at days 0, 7, 14, 28, and 42 (*n* = 4, F). Photographic images showing inflamed ankle joints at different time points (G). Scale bars: 3 mm. Harvested blood was subjected to a luciferase assay (H). (I and J) Seven days after treatment with PBS or rAAV9 carrying pCBA.mCherry or pIR.gCherry, 2-month-old WT mice were injected i.p. with arthritic K/BxN serum at days 0 and 2 and euthanized at day 12. GFP expression in AAV-treated hindlimbs and synovial tissues was assessed by IVIS optical imaging (*n* = 8–10, I) and qRT-PCR (*n* = 4–5, J), respectively. PBS was used as a negative control. Data are representative of two independent experiments (C and D). Values represent mean ± SD by one-way ANOVA test (B, E, and G–J).

Aberrant activation of IL-1 signaling is associated with RA and AIDs.^11^^,^^18^ Treating patients with anakinra has proven to be therapeutically effective for these diseases, but its daily administration presents a high burden to the patients who require long-term treatment.^34^ Given that inflammatory stimuli induce expression of sIL-1Ra as a negative feedback mechanism,^30^ we sought to develop the rAAV vector that produces secreted human IL-1Ra in response to inflammation to copy this naturally occurring therapeutic mechanism. RA patients show elevated levels of pro-inflammatory cytokines that activate the NF-κB pathway, such as TNF, IL-1β, IL-6, and IL-17,^36^ and bone morphogenic proteins (BMPs) 2, 4, and 6^37^^,^^38^ in synovial tissues, resulting in persistent inflammation and joint destruction. We therefore sought to construct a promoter that enables synergistic responses to these inflammatory signals (inflammation-responsive promoter [pIR]) by combining two promoters: (1) the mouse *Id1* promoter (−1,075/–993) containing two SMAD-binding elements (SBEs), the binding sites of YY1, EGR1, and CREB/ATF, and one BMP-responsive element (BRE)^39^ and (2) the *PB2* promoter containing two NF-κB-binding sites and one FosP-binding site.^40^ To reduce the size of the rAAV plasmid, the 1,027-bp chicken β-actin (CBA) intron was replaced with the 384-bp MassBiologics (MBL) intron^41^ (AAV.pIR; Figure 1A). Finally, three reporter genes—Gaussia luciferase (gLuc), enhanced green fluorescent protein (EGFP), and mCherry—were each separately cloned into the AAV.pIR plasmid to examine responsiveness to inflammatory cytokines and BMPs (Figure S1).

AAV.pIR.gLuc-expressing cells were stimulated with human TNF, IL-1β, or BMP4, and a significant, dose-dependent increase in luciferase activity was observed (Figure 1B). Luciferase activity increased further when cells were co-treated with BMP4 and either TNF or IL-1β (Figure S2A), demonstrating additive responsiveness to inflammatory cytokines and BMPs. Notably, the AAV.pIR.gLuc plasmid shows higher responsiveness to these stimuli than the benchmark reporter genes for NF-κB signaling (PB2-Luc) and BMP signaling (BRE-Luc) (Figures S2B and S2C). Since the rAAV9 serotype has been reported for its robust systemic transduction to organs such as liver, muscles,^42^^,^^43^^,^^44^ skeleton,^45^^,^^46^ and synovial tissue,^47^ we packaged the AAV plasmids encoding pIR.gLuc, pIR.EGFP, pIR.mCherry, pCBA.EGFP, and pCBA.mCherry into rAAV9 capsids (Figure S1). Primary calvarial osteoblasts (COBs) transduced with rAAV9.pIR-EGFP were cultured under non-differentiating growth (ND) or osteoblast-differentiating (OBD) conditions in the presence or absence of various inflammatory stimuli, including TNF, IL-1β, IL-17A, lipopolysaccharide (LPS), IL-6, IFN-γ, IL-22, and IL-23. rAAV9.pCBA.EGFP was used as a control for constitutive expression driven by the CBA promoter.^48^ GFP expression in AAV-transduced cells was assessed by immunoblotting with an anti-GFP antibody (Figure 1C) and fluorescence microscopy (Figure 1D). pCBA.EGFP-transduced cells showed high expression of GFP, which was comparable to that in pIR.EGFP-transduced cells after stimulation with TNF, TNF + IL-17A, LPS, or IL-1β. This was consistent with pIR.EGFP-transduced bone marrow-derived monocytes showing robust expression in the presence of RANK ligand (RANKL), TNF, TNF + IL-17A, LPS, or IL-1β (Figures S2D and S2E). However, only modest expression was observed in pIR.EGFP-transduced COBs under OBD conditions or when stimulated with non-NF-κB-activating inflammatory cytokines IL-17A, IL-6, IFN-γ, IL-22, and IL-23. These results suggest that the IR promoter can distinguish different inflammatory stimuli and induce transgene expression in response.

To examine dynamic responsiveness of the IR promoter to inflammatory stimuli *in vitro*, pIR.gLuc-transduced COBs were cultured with TNF or IL-1β for 3 days, then switched to cytokine-free medium. Cell supernatant was collected to measure the activity of secreted gLuc. Luciferase activity in the supernatant peaked at day 3 after the initial stimulation and gradually decreased to basal level after the removal of the cytokine (Figure 1E), demonstrating the IR promoter’s ability to perform ON and OFF responses to inflammatory cytokines. To test *in vivo* responsiveness of the IR promoter to inflammatory arthritis, we utilized the established mouse model of RA K/BxN serum transfer arthritis (STA). K/BxN STA mice develop spontaneous autoimmune arthritis that mimics human RA, with leukocyte invasion in joints, pannus formation, cartilage destruction, and bone erosion. Inflammation peaks 12 days after the initial injection of serum and resolves after 28 days.^49^^,^^50^ Wild-type (WT) mice were injected intravenously (i.v.) with rAAV9.pIR.gLuc 1 week prior to two intraperitoneal (i.p.) injections of K/BxN serum. Peripheral blood was collected by cheek bleeding at days 7, 14, 28, and 42 and prior to serum injection (day 0, Figure 1F). As previously described,^51^ joint inflammation was found to peak approximately 14 days post-initial injection of K/BxN serum (Figures S2F and S2G) and gradually decrease at 28 and 42 days (Figure 1G). The inflammation curve corresponded to increased luciferase activity in the blood at days 7 (6) and 14 (12), followed by a decrease at 28 and 42 days (Figures 1H and S2H), confirming the dynamic responsiveness of the IR promoter to inflammatory arthritis in mice.

To determine the degree of transgene expression driven by the IR promoter, we compared expression levels of mCherry in arthritic mice treated with rAAV9 carrying pIR.mCherry (inflammation-inducible expression) or pCBA.mCherry (constitutive expression). rAAV-treated mice were injected i.p. with K/BxN serum, and mCherry expression in the hindlimb muscle (Figure 1I) and ankle synovial tissue (Figure 1J) were assessed by optical imaging and quantitative PCR (qPCR), respectively. Unlike our *in vitro* culture data showing comparable expression levels in the cells transduced with pCBA.EGFP and pIR.EGFP after inflammatory stimulation, under RA conditions, expression levels in the muscle and synovial tissue of pIR.mCherry-transduced mice are substantially lower than those in pCBA.mCherry-transduced mice. The IR promoter appears to be reflective of physiologic expression levels, showing highly active but not constitutive transcription of the reporter gene under inflammatory conditions. Thus, rAAV9.pIR is a promising vector that can tightly control transgene expression in response to inflammation *in vitro* and *in vivo*. Nonetheless, since the pIR promoter also responds to various biological processes, including cell survival, proliferation, and metabolism, non-specific and excessive transgene expression during early skeletal development may lead to abnormal bone growth or other development abnormalities.

### AAV-mediated expression of human sIL-1Ra inhibits IL-1 signaling and inflammatory arthritis

**Figure 2:**
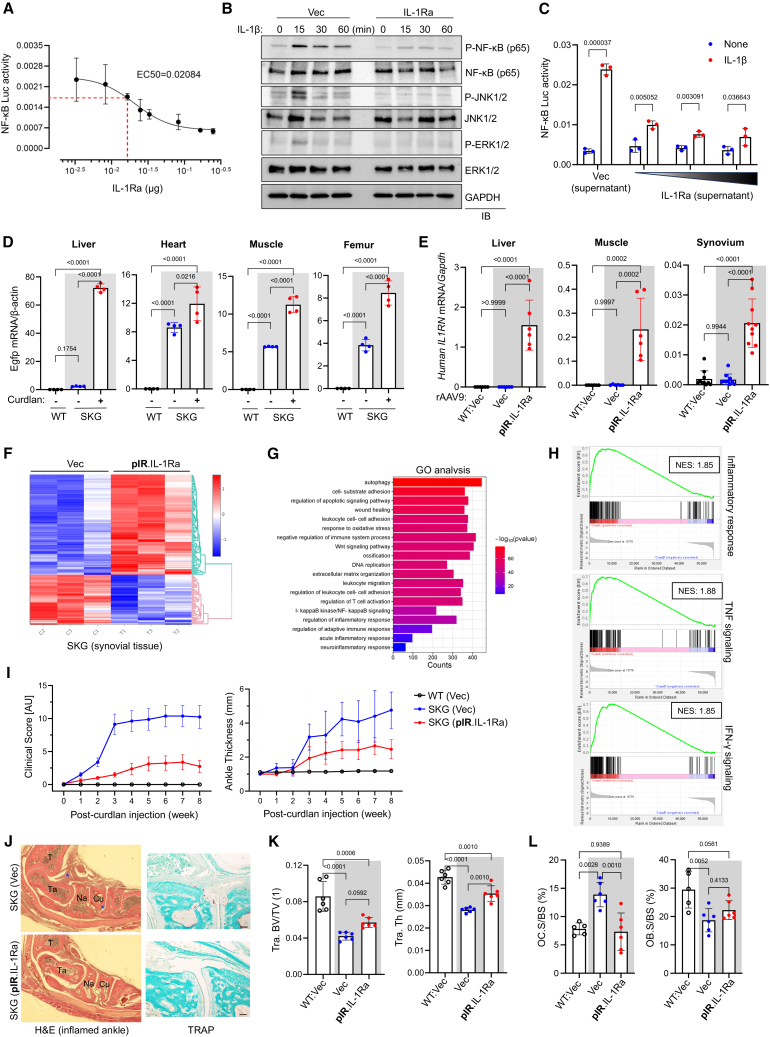
AAV-mediated expression of human sIL-1Ra attenuates IL-1 signaling, inflammation, and bone loss in arthritic SKG mice (A) HEK293 cells were transiently transfected with varying concentrations of the pCBA.IL-1Ra plasmid, along with PB2-Luc and *Renilla* reporter genes, and 24 h later, stimulated with human IL-1β. Luciferase activity was normalized to *Renilla* luciferase (*n* = 3). (B) Two days after transfection with vector control (Vec) plasmid or pCBA.IL-1Ra plasmid, HEK293 cells were stimulated with human IL-1β for 0, 15, 30, and 60 min and then, immunoblotted with the indicated antibodies. Anti-GAPDH antibody was used as a loading control. (C) HEK293 cells were transfected with Vec plasmid or pCBA.IL-1Ra plasmid, and the supernatant was collected 2 days post-transfection. Different volumes of the collected supernatant were incubated with the HEK293 cells transfected with PB2-Luc and *Renilla* reporter genes, in the absence or presence of human IL-1β (*n* = 3). (D) WT and SKG mice, 12 weeks old, were injected i.v. with rAAV9.pIR.EGFP 2 weeks prior to PBS or curdlan injection; 2 weeks later, GFP expression was measured in the indicated tissues by qPCR (*n* = 4). Gray boxes indicate SKG mice. (E–L) WT and SKG mice, 12 weeks old, were injected i.v. with rAAV9 carrying Vec or pIR.IL-1Ra 2 weeks prior to curdlan injection (*n* = 6). Ankle thickness and clinical joint inflammation were measured weekly (I). Six weeks later, expression of human sIL-1Ra (*IL1RN*) was assessed in the liver, muscle, and synovial tissues (E). Alternatively, total RNA isolated from synovial tissues was subjected to bulk RNA sequencing (*n* = 3). Heatmap shows differential gene expression in the SKG mice treated with Vec or pIR.IL-1Ra. The log_10_ expression (read count) was centered across samples. Red and purple denote upregulation and downregulation, respectively (F). Gene Ontology (GO) analysis reveals multiple biological processes associated with immune responses (G). Gene set enrichment analysis (GSEA) shows enrichment of genes involved in inflammatory response, TNF signaling, and IFN-γ signaling (H). H&E- and TRAP-stained sections of inflamed ankles demonstrate that pIR.IL-1Ra treatment protects from articular erosion in arthritic SKG mice (J). The asterisk indicates immune infiltrates. Scale bars: right, 100 μm. Cu, cuneiform; Na, navicular bone; T, tibia; Ta, talus (*n* = 6). MicroCT analysis showing femoral trabecular bone mass from AAV-treated WT and SKG mice (*n* = 6, K). Tra. BV/TV, trabecular bone volume/tissue volume; Tra.Th, trabecular thickness. Histologic quantification of osteoclasts and osteoblasts on the bone surface (*n* = 6, L). OB.S/BS, osteoblast surface/bone surface; OC.S/BS, osteoclast surface/bone surface. Gray boxes indicate SKG mice. Values represent mean ± SD by one-way ANOVA test (A, C–E, K, and L). Representative images of three replicates are displayed (B and J).

We next constructed an AAV plasmid expressing human sIL-1Ra (Figure S3A) and examined the ability of AAV to inhibit the activation of NF-κBs and MAPKs downstream of IL-1 signaling. HEK293 cells expressing the NF-κB-responsive reporter gene (NF-κB-Luc) were transfected with different doses of sIL-1Ra and then stimulated with human IL-1β. sIL-1Ra expression effectively suppressed luciferase activity in a dose-dependent manner (Figure 2A), which corresponded to a significant decrease in phosphorylation levels of the NF-κB subunit p65 (Figure 2B). IL-1β-induced phosphorylation of JNK1/2 and ERK1/2 MAPKs was also markedly reduced in these cells (Figure 2B), demonstrating that AAV-mediated expression of sIL-1Ra is a potent suppressor of IL-1 signaling. Because endogenous sIL-1Ra is a secreted ligand,^21^^,^^22^ we measured the protein levels of sIL-1Ra in the supernatant of transfected cells, demonstrating increased secretion in a dose-dependent manner (Figure S3B). Likewise, the supernatant containing sIL-1Ra also inhibited NF-κB luciferase activity following IL-1β treatment (Figure 2C). These results demonstrate that our rAAV vector producing secreted human IL-1Ra is a potent inhibitor of IL-1 signaling. Because neutralizing antibodies (nAbs) against rAAV capsid and human IL-1Ra could impact the therapeutic efficacy of AAVs, we measured serum levels of anti-rAAV9 nAbs and human IL-1Ra following a single i.v. injection of human IL-1Ra-expressing rAAV9 (2.5 × 10^13^ vector genomes [vg]/kg). As expected, high expression of anti-rAAV9 nAbs in peripheral blood was detected 4 weeks post-injection (Figure S3C). Despite the presence of anti-AAV9 nAbs, serum levels of human IL-1Ra remained unchanged over time, whereas nAbs against human IL-1Ra gradually increased starting at 4 weeks post-rAAV injection (Figures S3D–S3F).

To determine whether inflammation-inducible expression of human sIL-1Ra via systemic delivery of rAAV9 can ameliorate inflammatory arthritis, SKG mice were injected i.v. with rAAV9 carrying pIR.EGFP or pIR.IL-1Ra. SKG mice harbor a mutation in the *Zap-70* gene, resulting in abnormal T cell receptor signaling and increased numbers of peripheral autoreactive T cells.^52^^,^^53^ Onset of inflammation in SKG mice can be synchronized and augmented with systemic injection of 1,3-β-glucan (curdlan), which causes inflammatory arthritis in the joints; elevated levels of TNF, IL-17, IL-1, and IL-6; and osteoclast-mediated joint destruction (Figures S4A and S4B).^52^^,^^53^^,^^54^ To examine the IR promoter-driven expression patterns and biodistribution in arthritic SKG mice, inflammatory arthritis in these mice was boosted by curdlan treatment 2 weeks after i.v. injection of rAAV9.pIR.EGFP; 2 weeks later, mRNA and protein levels of GFP in individual tissues were assessed by quantitative real-time PCR (RT-qPCR), *in vivo* imaging system (IVIS) optical imaging, and fluorescence microscopy on cryo-sectioned tissues (Figures 2D and S4C–S4F). Without curdlan injection, these mice showed a modest expression in liver, heart, muscle, and femur, with little expression in brain tissue. However, the expression of AAV in all assessed tissues was substantially increased following curdlan injection. Notably, the expression was also detected in synovial tissues and calcaneus bones, but not in the articular cartilage, within inflamed ankle joints (Figure S4E). These results demonstrate that the IR promoter-driven expression via systemic delivery of rAAV9 is robustly responsive to inflammatory arthritis in the SKG mouse model of RA. Next, we treated arthritic SKG mice with an i.v. injection of rAAV9.pIR.IL-1Ra to test whether the IR promoter-driven expression of human sIL-1Ra could ameliorate inflammatory arthritis. Similar to pIR.EGFP-treated SKG mice, these mice showed a high expression of human sIL-1Ra (*ILRN1*) in the liver, muscle, and synovial tissue after curdlan injection (Figure 2E). In concurrence with increased sIL-1Ra expression, mRNA levels of *Il1b* and its downstream genes *Il1rn* and *Il6* were markedly decreased in synovial tissue (Figure S5A), indicating the ability of rAAV9.pIR.IL-1Ra to inhibit IL-1 signaling *in vivo*. Whole-transcriptome analysis revealed a stark contrast of gene expression patterns in the synovial tissues between mice treated with vector control (Vec) and pIR.IL-1Ra (Figure 2F). These differentially expressed genes (DEGs) are associated with numerous biological processes, including autophagy, apoptosis, wound healing, oxidative stress, ossification/extracellular matrix formation, and inflammatory responses (Figure 2G). Similarly, the gene set enrichment analysis (GSEA) indicated high enrichment of genes associated with inflammatory responses, TNF signaling, and IFN-γ signaling (Figure 2H). These results demonstrate that the IR promoter-driven expression of sIL-1Ra via systemic delivery of rAAV9 can potentially inhibit gene transcription associated with IL-1 signaling and inflammatory arthritis in arthritic SKG mice.

To examine the ability of rAAV9.pIR.IL-1Ra to inhibit inflammatory arthritis, clinical inflammation and ankle thickness of pIR.sIL-1Ra-treated SKG mice after curdlan injection were scored, demonstrating the potency of AAVs to suppress joint inflammation (Figures 2I and S5B). This is consistent with a histology analysis showing a significant decrease in the degree of inflammatory infiltrates in inflamed ankle joints (Figure 2J, left). As joint inflammation was reduced by pIR.sIL-1Ra treatment, the number of tartrate-resistant acid phosphatase-positive (TRAP^+^) osteoclasts and bone erosion pits in inflamed ankle joints also markedly decreased (Figures 2J, right, and S5C). Thus, the IR promoter-driven expression of human sIL-1Ra via systemic delivery of rAAV9 can protect articular bone from the erosions that result from joint inflammation in this model of RA. In addition to joint inflammation, systemic bone loss in arthritic SKG mice is partly reversed by rAAV9.pIR.IL-1Ra treatment, as evidenced by increased trabecular bone mass and cortical bone density in femurs (Figures 2K and S5D). Compared to WT femurs, SKG femurs show an increased number of TRAP^+^ osteoclasts, which was reversed by pIR.IL-1Ra treatment. Conversely, the number of osteoblasts in SKG femurs was markedly decreased compared to WT femurs, but it was not reversed by pIR.IL-1Ra treatment (Figure 2L). These results demonstrate that rAAV9.pIR.IL-1Ra limits both articular bone erosion and systemic bone loss in arthritic SKG mice via suppression of osteoclast development. Thus, inflammation-inducible expression of human sIL-1Ra via systemic delivery of rAAV9 is a promising approach to suppress inflammation and bone loss in the SKG mouse model of RA.

### Inflammation-inducible expression of human sIL-1Ra ameliorates inflammatory arthritis in K/BxN STA mice

**Figure 3:**
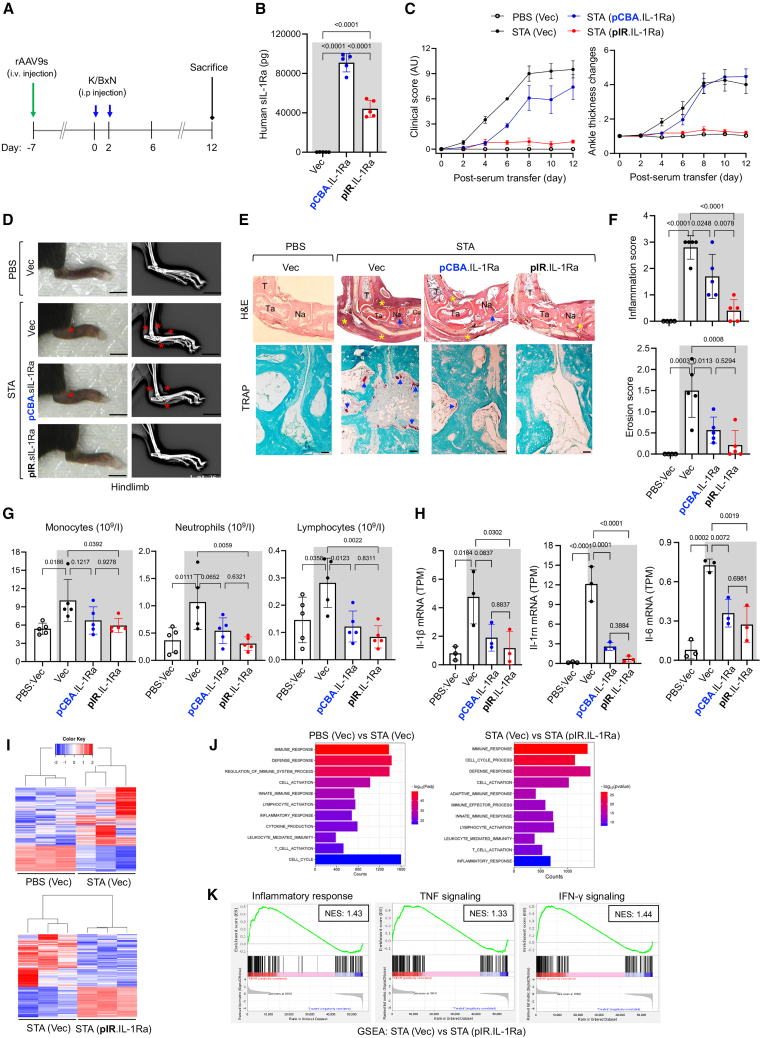
AAV-mediated expression of human sIL-1Ra inhibits inflammatory arthritis in K/BxN STA mice (A) Diagram of the study and treatment methods. Two-month-old mice were injected i.p. with PBS or arthritic K/BxN serum at days 0 and 2 after i.v. injection of rAAV9 carrying Vec, pCBA.IL-1Ra, or pIR.IL-1Ra (*n* = 5). (B) Protein levels of human sIL-1Ra in blood were measured by ELISA (*n* = 5). (C) Clinical inflammation and ankle thickness scores were measured weekly, showing a stronger suppressive effect of pIR.IL-1Ra compared to pCBA.IL-1Ra (*n* = 5). (D) Representative photographic and radiographic images showing ankle joints of AAV-treated mice. Scale bars: 3 mm. The asterisk indicates inflamed ankle joints. (E and F) H&E- and TRAP-stained sections of inflamed ankles (E) and relative quantification of inflammation and erosion (*n* = 5, F), demonstrating that human sIL-1Ra expression protects against articular erosion. Scale bars: (E) bottom, 50 μm. The asterisk indicates immune cell infiltrates. Arrows indicate TRAP^+^ osteoclasts and bone erosion pits. (G) Complete blood count (CBC) test demonstrates a significant decrease in monocytes, neutrophils, and lymphocytes in peripheral blood following treatment with pCBA.IL-1Ra or pIR.IL-1Ra (*n* = 5). Gray boxes indicate arthritic K/BxN STA mice. (H–K) Total RNA isolated from synovial tissues obtained from the Vec-treated mice (PBS [Vec] vs. STA [Vec]) or K/BxN STA mice (STA [Vec] vs. STA [pIR.IL-1Ra]) was subjected to bulk RNA sequencing (*n* = 3). TPM represents transcript numbers of IL-1-responsive genes. TPM, transcripts per million (H). Heatmap showing differential gene expression in PBS (Vec) vs. STA (Vec) or STA (Vec) vs. STA (pIR.IL-1Ra). The log_10_ expression (read count) was centered across samples. Red and purple denote upregulation and downregulation, respectively (I). GO analysis shows multiple biological processes associated with immune responses (J). GSEA analysis shows enrichment of genes involved in inflammatory response, TNF signaling, and IFN-γ signaling (K). Gray boxes indicate arthritic K/BxN STA mice. Values represent mean ± SD by one-way ANOVA test (B, C, and F–H). Representative images of five replicates are displayed (D and E).

To directly test the ability of AAV to suppress IL-1-driven inflammatory arthritis in mice, we utilized the K/BxN STA mouse model of RA. WT mice were injected i.p. with K/BxN serum to induce RA 7 days after i.v. injection of rAAV9 carrying Vec, pCBA.IL-1Ra, or pIR.IL-1Ra (Figure 3A). Protein levels of sIL-1Ra in peripheral blood were measured by enzyme-linked immunosorbent assay (ELISA), revealing higher circulating sIL-1Ra in mice treated with pCBA.IL-1R (constitutive expression) relative to pIR.IL-1Ra (inflammation-inducible expression; Figure 3B). Clinical inflammation and ankle thickness scores demonstrate that joint inflammation peaked 12 days post-initial injection of serum in Vec-treated arthritic mice. Interestingly, joint inflammation was modestly decreased by pCBA.IL-1Ra treatment, but almost completely suppressed when treated with pIR.IL-1Ra (Figures 3C and 3D), indicating that more expression did not produce a better therapeutic outcome. Similarly, compared to the arthritic Vec-treated mice, pCBA.IL-1Ra-treated mice show a modest reduction in inflammatory infiltrates into ankle joints, but they were completely suppressed by pIR.IL-1Ra treatment (Figures 3E and 3F, top). This was accompanied with TRAP staining analysis of inflamed ankle joints showing a significant decrease in the number of TRAP^+^ osteoclasts and bone erosion pits in the arthritic mice treated with pIR.IL-1Ra relative to pCBA.IL-1Ra or Vec (Figures 3E and 3F, bottom). However, the decrease in the numbers of monocytes, neutrophils, and lymphocytes in peripheral blood was comparable between the arthritic mice treated with pCBA.IL-1Ra and pIR.IL-1Ra, suggesting that constitutive and inflammation-inducible expressions of sIL-1Ra are both potent to suppress systemic inflammation (Figure 3G). Notably, red blood cells (RBCs) and hemoglobin levels in peripheral blood were unaffected in these mice, indicating little effect on erythropoiesis (Figure S6A). These results demonstrate that inflammation-inducible expression of human sIL-1Ra is more effective than constitutive expression in suppressing joint inflammation and articular bone erosion in the K/BxN STA mouse model of RA.

To gain insight into inflammation-inducible expression of human sIL-1Ra on molecular pathways associated with inflammatory arthritis in RA, ankle synovial tissues were isolated from Vec-treated healthy mice and Vec- or pIR.IL-1Ra-treated STA mice and then subjected to bulk RNA sequencing (RNA-seq). As expected, the expression of *Il1b* and its downstream genes *Il1rn* and *Il6* was markedly upregulated in Vec-treated arthritic mice relative to Vec-treated healthy mice, but it was almost completely reversed by pCBA.IL-1Ra or pIR.IL-1Ra treatment (Figure 3H). These results further confirmed the ability of rAAV9.pIR.IL-1Ra to inhibit IL-1 signaling in mouse models of RA. In line with this, whole-transcriptome analysis indicated a substantial alteration in gene expression profiles under arthritic conditions relative to healthy conditions. Remarkably, this alteration was largely reversed by pIR.IL-1Ra treatment (Figure 3I). For example, a volcano plot comparing Vec-treated healthy mice and Vec-treated STA mice identified numerous DEGs (downregulated: 1,050; upregulated: 460), whereas the number of DEGs between Vec-treated healthy mice and pIR.IL-1Ra-treated STA mice was significantly low (downregulated: 72; upregulated: 79) (Figure S6B). Gene Ontology analysis shows that these genes are highly enriched in several biological processes, including cell cycle and activation, inflammatory, innate, and adaptive immune responses, and lymphocyte and leukocyte activation (Figure 3J), and the molecular pathways associated with inflammatory arthritis, including inflammatory responses, TNF signaling, and IFN-γ signaling (Figure 3K). These results demonstrate that inflammation-inducible expression of human sIL-1Ra effectively inhibits IL-1-driven inflammatory signaling in K/BxN STA mice. Inflammation-inducible expression of human sIL-1Ra via systemic delivery of rAAV9 is a promising therapeutic option for blocking IL-1-mediated inflammation and bone destruction to combat RA pathology. As *in vivo* expression of rAAV typically requires approximately 5 days following i.v. injection, and K/BxN STA mice reach peak inflammation around 12 days after serum transfer, this RA model was used to evaluate the prophylactic effects of our AAV gene therapy rather than therapeutic effects. The widely used CIA mouse model could further enhance the translational relevance of these findings by enabling the evaluation of AAV-based therapeutic interventions.

### AAV-mediated expression of human sIL-1Ra prevents systemic inflammation in a mouse model of DIRA

DIRA (OMIM: 612852) is an ultra-rare genetic disorder characterized by neonatal onset of autoinflammatory symptoms, including severe skin and bone inflammation, joint swelling, and intense pain. Patients with DIRA have autosomal recessive mutations in the *IL1RN* gene. Lack of IL-1Ra production in these patients results in excessive IL-1 production due to dysregulation of IL-1 signaling, leading to systemic autoinflammation that can cause life-threatening multi-organ failure.^14^ Although anakinra has proven to be effective for treating patients with DIRA,^14^ its daily subcutaneous injection is not optimal for patients who require lifelong treatment. Since rAAV-based gene therapy holds promise for providing durable treatments for many genetic disorders,^35^ sIL-1Ra-expressing rAAV vector may be a plausible therapeutic strategy for DIRA.

**Figure 4:**
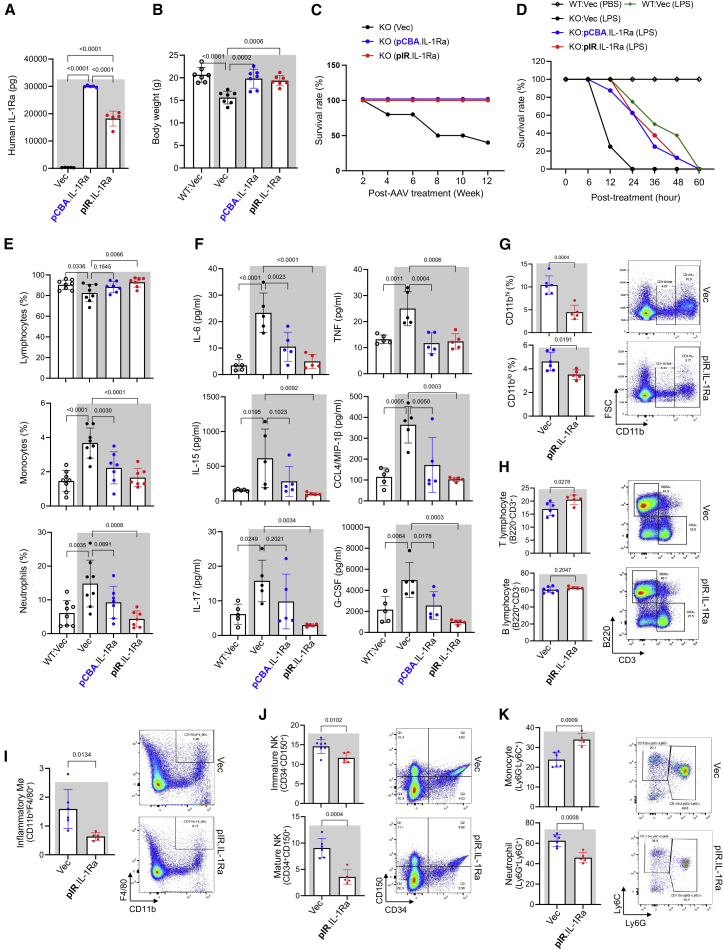
AAV-mediated expression of human sIL-1Ra reverses systemic inflammation in DIRA mice Three-week-old WT (*Il1rn*^*+/+*^) and KO (*Il1rn*^−/−^) mice were injected i.v. with rAAV9 carrying Vec, pCBA.IL-1Ra, or pIR.IL-1Ra. (A and B) Ten weeks post-injection, protein levels of human IL-1Ra in the blood (A) and body weight (B) were measured (*n* = 7). (C) Survival rates of AAV-treated mice were monitored biweekly until the age of 12 weeks (*n* = 7). (D) Three-week-old WT and KO mice were injected i.v. with rAAV9s, and 4 weeks later, they were challenged with an i.p. injection of 10 mg/kg LPS (*n* = 10). Survival was monitored every 6 h, demonstrating that sIL-1Ra-treated KO mice become more resistant to septic shock. (E–K) Peripheral blood was harvested from WT and KO mice 10 weeks after AAV injections and then subjected to CBC test, demonstrating a reversal of monocytes, neutrophils, and lymphocytes in KO mice after treatment with pCBA.IL-1Ra or pIR.IL-1Ra (E, *n* = 8). Protein levels of inflammatory cytokines and chemokines in the peripheral blood of KO mice were decreased by treatment with pCBA.IL-1Ra or pIR.IL-1Ra (F, *n* = 5). Immune cells were isolated from the spleens of Vec- or pIR.IL-1Ra-treated KO mice and analyzed by flow cytometry (G–K, *n* = 6). The *y* axis indicates frequency of cell populations (%). Dot plots represent relative frequency of CD11b^high^ (left, top) and CD11b^low^ (left, bottom) populations (G, left). Gray boxes indicate KO mice. Values represent mean ± SD by a two-tailed unpaired Student’s t test for comparing two groups (G–K) or one-way ANOVA test (A, B, E, and F).

IL-1Ra-deficient mice (*Il1rn*^−/−^)^33^^,^^55^ were used as a mouse model of DIRA to test the therapeutic effects of our rAAV vector. We confirmed deletion of the *Il1rn* gene in these mice by measuring mRNA and protein levels (Figures S7A and S7B). Three-week-old WT (*Il1rn*^+/+^) and knockout (KO) (*Il1rn*^−/−^) mice were treated with a single i.v. injection of rAAV9 carrying Vec, pCBA.IL-1Ra, or pIR.IL-1Ra, and 10 weeks later, human sIL-1Ra expression in peripheral blood, liver, and heart was assessed (Figures 4A and S7C). While pIR.IL-1Ra-treated mice showed significantly lower expression of sIL-1Ra in the liver than pCBA.IL-1Ra-treated mice, the difference in expression was more modest in peripheral blood and comparable in the heart (Figures 4A and S7D). These results demonstrate differential expression (DE) patterns of human sIL-1Ra in different organs of rAAV-treated mice. Compared to Vec-treated WT mice, Vec-treated KO mice show a decrease in body weight (Figure 4B) and survival rates (Figure 4C) and poor locomotion (Video S1). These phenotypes were fully reversed by a single i.v. injection of rAAV9 vector carrying pCBA.IL-1Ra or pIR.IL-1Ra, suggesting that human sIL-1Ra-expressing rAAV9 is effective in improving body metabolism, locomotion, and survival of KO mice by limiting systemic inflammation. However, AAV treatment has no grossly apparent effect on non-skeletal tissues, including heart, lung, liver, spleen, and kidney, in KO mice (Figure S7E) and total numbers of white blood cells (WBCs), RBCs, and platelets (PLTs) are also normal (Figure S7F). These results suggest that pIR promoter-driven expression of human sIL-1Ra via systemic rAAV9 can limit untoward off-target effects.

Video S1. Three-week-old WT (Il-1rn^+/+^) and KO (Il-1rn^–/–^ ) mice were injected i.v. with rAAV9 carrying vector control (Vec) or pIR.IL-1Ra, and mouse behaviors were assessed 10 weeks postinjection (*n* = 5) Movie shows the body size and mobility of 13-week-old AAV-treated mice, including a Vec-treated WT mouse (normal body size, mobility, and activity level), a Vec-treated KO mouse (reduced body size, mobility, and activity level), and a pIR.IL-1Ra-treated KO mouse (normal body size, mobility, and activity level). The arrow indicates the pIR.IL-1Ra-treated KO mouse.

Because KO mice are more susceptible than WT mice to the lethal effects of endotoxin,^56^ 3-week-old WT and KO mice were treated with rAAV injections, and then 4 weeks later, they were challenged by i.p. injection of LPS. Following LPS injection, WT mice typically survive up to 60 h, whereas KO mice die within 24 h. However, survival in KO was markedly improved after treatment with pCBA.IL-1Ra or pIR.IL-1Ra (Figure 4D). This result indicates that rAAV treatment enhances the resistance of KO mice to septic shock. Similarly, compared to WT mice, KO mice show increased numbers of monocytes and neutrophils as well as decreased numbers of lymphocytes in the peripheral blood, which is also reversed by treatment with pCBA.IL-1Ra or pIR.IL-1Ra (Figure 4E). This is accompanied by elevated levels of circulating blood cytokines and chemokines associated with inflammation, including TNF, IL-6, IL-15, IL-17, CCL4, and granulocyte-colony-stimulating factor (G-CSF) in KO mice (Figure 4F), while there was little to no alteration in inflammatory cytokines, chemokines, and growth factors, including leukemia inhibitory factor (LIF), macrophage-CSF (M-CSF), GM-CSF, IFN-γ, IL-2, IL-3, IL-4, IL-5, IL-7, IL-9, IL-10, IL-12, IL- 13, C-X-C motif chemokine ligand 1 (CXCL-1), CXCL-2, CXCL-5, CXCL-9, CXCL-10, CCL-2, CCL-3, CCL-5, CCL-11, and vascular endothelial growth factor-A (VEGF-A) (Figure S8). Notably, compared to Vec-treated WT spleen, Vec-treated KO spleen showed a significant increase in IL-1α and IL-1β expression, but expression levels in peripheral blood, bone, and joints^33^ were comparable between WT and KO mice, suggesting tissue-specific differences in IL-1 signaling activity in mice (Figures S8A and S8B). Thus, systemic delivery of human sIL-1Ra-expressing rAAV9 is a powerful approach to downregulate circulating immune cells and inflammatory cytokines and chemokines in the blood of KO mice.

Finally, flow cytometry analysis of splenocytes was performed to assess the effects of rAAVs on the development of immune cells and hematopoietic stem cells in KO mice. pIR.IL-1Ra treatment resulted in decreased numbers of CD11b^+^ myeloid cells and increased numbers of CD3^+^ T lymphocytes in the spleen (Figures 4G and 4H). Specifically, these splenocytes showed a significant decrease in immune cell populations, including inflammatory macrophages, immature and mature natural killer cells, and mature neutrophils, an increase in immature monocytes, and minimal changes in B lymphocytes (Figures 4I–4K). These results demonstrate that AAV-mediated expression of human sIL-1Ra effectively lowers circulating inflammatory myeloid cell populations in the periphery of KO mice while increasing peripheral immature monocytes and T lymphocytes. Taken together, our data indicate that systemically delivered rAAV9.pIR.IL-1Ra was able to effectively produce human sIL-1Ra in multiple tissues and ameliorate systemic inflammation in KO mice via a reversal of circulating inflammatory cytokines and chemokines and peripheral immune cell populations.

### AAV-mediated expression of human sIL-1Ra reverses skeletal phenotypes in a mouse model of DIRA

**Figure 5:**
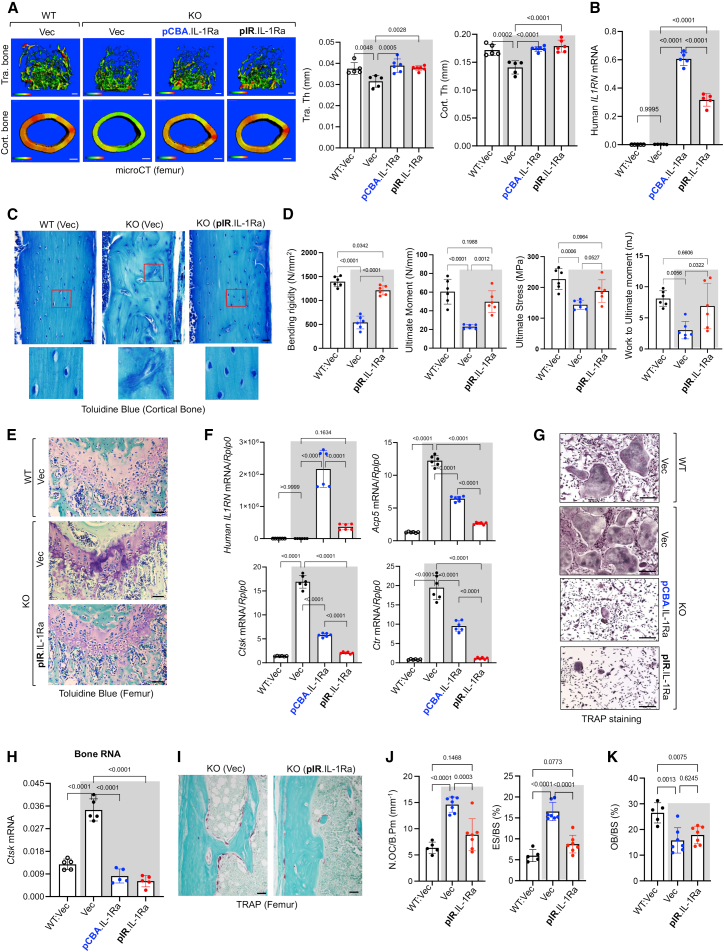
AAV-mediated expression of human sIL-1Ra reverses skeletal phenotypes of DIRA mice Three-week-old WT (*Il1rn*^*+/+*^) and KO (*Il1rn*^−/−^) mice were injected i.v. with rAAV9 carrying Vec, pCBA.IL-1Ra, or pIR.IL-1Ra. (A) MicroCT analysis was performed 10 weeks post-injection. At left, 3D reconstruction and relative quantification of trabecular and cortical bone thickness shows reversal of bone loss in IL-1Ra-treated KO femurs (*n* = 5). Scale bars: 200 μm. (B) qPCR analysis shows mRNA levels of human sIL-1Ra in the tibia (*n* = 5). (C and E) Toluidine blue-stained femoral sections show reversal of disorganized mineral and collagen architecture in the cortical bone (C) and growth plate columns (E) in sIL-1Ra-treated KO mice. Scale bars: 100 μm (C) and 50 μm (E). (D) Biomechanical properties of rAAV-treated WT and KO femurs, including bending rigidity, ultimate moment, ultimate stress, and work to ultimate moment were quantified (*n* = 6). (F and G) Primary WT and KO BMMs were transduced with rAAV9 carrying Vec, pCBA.IL-1Ra, or pIR.IL-1Ra and then cultured in the presence of M-CSF and RANK ligand to differentiate into mature osteoclasts. mRNA levels of human IL-1Ra and osteoclastic genes (F) and TRAP-stained, multinucleated osteoclasts (G) were examined (*n* = 6). Scale bars: 100 μm. (H–K) Total RNA was isolated from WT and KO tibias 10 weeks after AAV injections, demonstrating a reversal of *Ctsk* expression in KO mice after treatment with pCBA.IL-1Ra or pIR.IL-1Ra (H, *n* = 5). TRAP-stained sections of Vec-treated WT femurs and Vec- or pIR.IL-1Ra-treated KO femurs (I) and relative quantification of osteoclasts (N.OC/B.Pm, number of osteoclast/bone perimeter), erosion surface (ES/BS, erosion surface/bone surface, J), and osteoblasts (OB.S/BS, osteoblast surface/bone surface, K) are displayed (*n* = 7). Scale bars: 100 μm. Gray boxes indicate KO mice. Values represent mean ± SD by one-way ANOVA test (A, B, D, F, H, J, and K) for multiple group comparisons. Representative images of six to seven replicates are displayed (A, C, E, G, and I).

DIRA patients have been reported to experience abnormal bone growth, deformities in the long bones, and severe bone inflammation,^14^ but these phenotypes are not observed in KO mice. Instead, these mice show normal structures of the skeleton, including long bones, skull, vertebrae, and joints (Figure S9A), but a significant reduction in femoral bone mass, mineral density, and mechanical strength, along with short stature (Figures 5A–5C, S9A, and S9B). Because the rAAV9 serotype is highly effective for the transduction of bone-residing cells, osteoblasts, and osteoclasts *in vitro* and *in vivo*,^45^^,^^46^ 3-week-old WT and KO mice were injected i.v. with rAAV9 carrying Vec, pCBA.IL-1Ra, or pIR.IL-1Ra. Ten weeks later, expression of human sIL-1Ra in the tibial bone was examined, demonstrating robust expression in pCBA.IL-1Ra-treated mice and slightly lower expression in pIR.IL-1Ra-treated mice (Figure 5B). Compared to Vec-treated WT mice, Vec-treated KO mice showed a significant decrease in trabecular and cortical bone thickness and cortical bone moment of inertia (MOI) and maximum MOI (Imax) in the femur, which corresponded to increased porosity of cortical bone (Figures 5A and S9B). This is consistent with histology showing disorganized mineral and collagen structure in KO femurs (Figure 5C). Remarkably, all of these skeletal abnormalities are reversed by treatment with pIR.IL-1Ra. Likewise, biomechanical tests of rAAV-treated femurs reveal that pIR.IL-1Ra treatment significantly improved the bone strength of KO mice, as shown by greater bending rigidity, ultimate moment, ultimate stress, and work to ultimate moment (Figure 5D). KO mice also exhibited a short stature where columns of proliferating and hypertrophic chondrocytes in the growth plate are disarranged, which is similar to those seen in the mice with constitutive activation of the NLRP3 inflammasome resembling NOMID.^11^^,^^57^ This phenotype was reversed by pIR.IL-1Ra treatment (Figure 5E). Thus, human sIL-1Ra-expressing rAAV9 vectors are effective in reversing skeletal abnormalities in KO mice, including low bone mass and strength and abnormal bone structure and growth plate.

Because IL-1 signaling induces bone erosion in RA via activation of osteoclasts,^58^ we hypothesized that persistent activation of IL-1 signaling by a lack of sIL-1Ra increases osteoclast differentiation, resulting in low bone mass and strength in KO mice. To test this, WT and KO bone marrow monocytes (BMMs) were differentiated into mature osteoclasts in the presence of M-CSF and RANKL after treatment with rAAV9 carrying Vec, pCBA.IL-1Ra, or pIR.IL-1Ra. As expected, osteoclast differentiation was substantially increased in Vec-treated KO BMMs relative to Vec-treated WT BMMs, as indicated by increased expression of the osteoclast genes, including *Acid phosphatase 5* (*Acp5*), *Cathepsin K* (*Ctsk*), and *Calcitonin receptor* (*Ctr*) (Figure 5F), and numbers of multi-nucleated osteoclasts (Figure 5G). The enhanced osteoclast development by DIRA was completely reversed by treatment with pCBA.IL-1Ra or pIR.IL-1Ra. Notably, mRNA levels of human sIL-1Ra (*IL1RN*) in pIR.IL-1Ra-treated BMMs were significantly lower than those in pCBA.IL-1Ra-treated BMMs, but this level of expression was sufficient to suppress osteoclast development (Figure 5F). Similarly, compared to WT femurs, KO femurs show a significant increase in the expression of the osteoclast gene *Ctsk* in bone RNA (Figure 5H), number of TRAP^+^ osteoclasts, and erosion surface of bone (Figures 5I and 5J), which was reversed by treatment with pIR.IL-1Ra. These results demonstrate that AAV-mediated expression of human sIL-1Ra is a potent suppressor of aberrant osteoclast development and osteoclast-mediated bone erosion in KO mice. Of note, osteoblast numbers on the surface of KO femurs were decreased compared to WT femurs, but human sIL-1Ra expression was unable to reverse this phenotype (Figure 5K), suggesting minimal effects of IL-1 signaling on osteoblast development. Our inflammation-inducible rAAV9, which produces circulating human sIL-1Ra by copying the pathophysiologic mechanism of endogenous IL-1Ra, represents a promising therapeutic approach for treating systemic inflammation and bone destruction in chronic inflammatory diseases.

## Discussion

The IL-1 pathway plays a crucial role in the pathogenesis of chronic inflammatory diseases such as RA and AIDs. Inhibition of IL-1 signaling by IL-1 blockers has proven therapeutically effective in these diseases.^18^^,^^34^ Moreover, the success and favorable safety profile of IL-1 blockers has led to their use in other AIDs, such as familial Mediterranean fever,^59^ TNF receptor-associated periodic syndrome,^60^ and hyperimmunoglobulinemia D with periodic fever syndrome.^61^ IL-1 blockers have been also encouraged to use for Still disease,^62^ Behcet disease, Schnitzler syndrome, and systemic juvenile idiopathic arthritis,^63^ which lack well-defined genetic etiologies. Finally, the accumulation of metabolic substrates, such as monosodium urate, ceramide, cholesterol, and glucose, activates the NLRP3-based inflammasome, triggering IL-1-mediated inflammation.^64^ This provides a strong rationale for targeting the IL-1 pathway to treat prevalent inflammatory diseases such as osteoarthritis,^65^ gout, diabetes mellitus, and coronary artery disease.^66^

Currently, three IL-1 blockers are approved for treating patients with RA or AIDs. Anakinra is a recombinant human IL-1Ra (rhIL-1Ra) that inhibits both IL-1α and IL-1β binding to the IL-1 receptor and is delivered by daily subcutaneous injections.^67^ Rilonacept is a dimeric Fc fragment containing the extracellular residues of IL-1R1 and IL-1RAcP that inhibits both IL-1α and IL-1β binding to the IL-1 receptor and is delivered by weekly subcutaneous injections.^68^ Canakinumab is a fully humanized anti-IL-1β monoclonal antibody that selectively inhibits IL-1β and is delivered by monthly subcutaneous injections.^69^ However, these short-acting agents impose a high treatment burden on RA patients with disability who need continuous long-term treatment. Additionally, these agents are not ideal for the treatment of the patients with AIDs who develop early onset of life-threatening autoinflammatory symptoms and require lifelong treatment. For example, DIRA treatment is complicated by the neonatal onset of skin and bone inflammation pathologies, such as pustular rash, the widening of ribs, periosteal reaction, multifocal osteolytic reactions, cervical vertebral fusion, hepatosplenomegaly, and multifocal osteomyelitis.^14^ Although anakinra is US Food and Drug Administration approved for treating DIRA, it requires lifelong daily subcutaneous administration, starting 1 week after the birth, which is challenging for parents, caregivers, and patients. Thus, an unmet need remains for developing IL-1 blockers that provide long-lasting therapeutic effects with a single administration, addressing accessibility challenges and alleviating treatment burden for patients.

Given that gene therapy using rAAV vectors holds promise for treating many genetic disorders,^35^ AAV gene therapy may be a plausible therapeutic strategy for DIRA. rAAVs have demonstrated high transduction efficiencies to brain, liver, muscle, heart, skin, and bone tissues to produce durable, long-term expression of therapeutic genes.^70^ Of note, rAAVs have been evaluated in over 145 clinical trials and more than 2,000 patients worldwide.^71^ The naturally occurring protein anakinra is likely to be more suitable for rAAV-mediated expression than rilonacept or canakinumab, which could be recognized as foreign antigens by the host immune system and neutralized by antibodies against them. However, two key characters of sIL-1Ra should be considered for the development of AAV gene therapy. First, the balance between sIL-1Ra and IL-1α/β is crucial to prevent the pathogenesis of inflammatory diseases while protecting against pathogens and maintaining cellular homeostasis. Second, unlike exogenous administration of anakinra, endogenous sIL-1Ra expression is tightly regulated via a negative feedback mechanism with inflammatory stimulation.

This study developed an rAAV gene therapy that can harness the pathophysiologic mechanism of endogenous sIL-1Ra in RA by producing secreted codon-optimized human IL-1Ra in response to pro-inflammatory cytokines and BMPs, which are enriched in the inflamed joints of RA patients. To test the ability of rAAV to ameliorate inflammatory arthritis in RA, we used SKG and K/BxN STA mice that have distinct pathogenic mechanisms, autoreactive T cell- and autoantibodies-driven RA, respectively. Prophylactic treatment of these mice with a single dose of rAAV9.pIR.IL-1Ra not only inhibits aberrant IL-1 signaling and osteoclast development but also ameliorates systemic and joint inflammation, systemic bone loss, and articular bone erosions. By contrast, supraphysiological and constitutive expression of human sIL-1Ra by rAAV9.pCBA.IL-1Ra treatment shows modest effects in these mice. This suggests that inflammation-induced expression of human IL-1Ra confers greater protection against inflammatory arthritis than constant, unregulated expression. Continuous high-level expression may increase immunogenicity, disrupt normal immune function, or cause IL-1 receptor desensitization. Consistently, a study using a mouse model of CIA showed that inflammation-inducible expression of human IL-1Ra driven by the C3-Tat/HIV promoter was more effective at suppressing inflammatory arthritis than constitutive expression driven by the cytomegalovirus (CMV) promoter.^72^ This suggests that the natural inflammatory response can be leveraged to induce on-demand sIL-1Ra production, offering greater therapeutic benefit than constant, potentially unnecessary expression.

Similar to the patients with DIRA harboring loss-of-function mutations in the *IL1RN* gene, KO (*Il1rn*^−/−^) mice develop early onset of systemic inflammatory arthritis due to dysregulated activation of IL-1 signaling. Expression of human sIL-1Ra was successfully reconstituted in multiple organs of KO mice when the rAAV9 carrying pCBA.IL-1Ra or pIR.IL-1Ra was injected i.v. into 3-week-old KO mice in which inflammatory processes are already active. AAV treatment improves body metabolism, survival rates, resistance to septic shock, and bone mass and strength by suppressing systemic inflammation and aberrant osteoclast-mediated bone destruction. These findings suggest that both constitutive and inflammation-inducible expressions of human sIL-1Ra are effective treatment strategies for DIRA. Moreover, systemic delivery of human sIL-1Ra-expressing rAAV9s is capable of both prophylactic and therapeutic improvements in mice with inflammatory arthritis. Further studies will be necessary to investigate the mechanisms responsible for the discrepancy of constitutive and inflammation-inducible expression of human sIL-1Ra in suppressing inflammatory arthritis in the mouse models of RA (K/BxN STA) and DIRA (*Il1rn*^−/−^).

Given that systemic administration of rAAV9 effectively transduces bone-residing cells, including osteoclasts, osteoblasts, and osteocytes, constitutive and supraphysiological expression of human sIL-1Ra in the skeleton via rAAV9 could lead to abnormal skeletal development and other developmental issues in early childhood of patients with AIDs. Accordingly, long-term treatment of patients with RA or AIDs with anakinra has been reported to cause untoward side effects, such as serious infections, neutropenia, and allergic reactions. Additionally, the impact of systemically delivered rAAV9 capsid on therapeutic outcomes in inflammatory arthritis is an important consideration, as AAV administration can itself trigger humoral immune responses—such as the generation of nAbs against the AAV capsid—as well as T cell-mediated cytotoxicity and TLR-mediated innate immunity. Thus, sIL-1Ra expression should be limited to inflammatory conditions, similar to naturally occurring pathophysiologic mechanisms. The rAAV9.pIR.IL-1Ra that produces secreted human IL-1Ra in response to inflammation mimics this mechanism. Further investigation for long-term therapeutic outcomes and physiological expression levels of human sIL-1Ra by systemic delivery of rAAV9.pIR.IL-1Ra would strengthen the translational relevance of this study. Furthermore, rAAV vector improvements such as capsid modification can also improve rAAV vector targeting to inflamed tissues in RA to produce more precise inhibition of aberrant IL-1 signaling in synovial fibroblasts and immune cells. Finally, future investigation for vector biodistribution, toxicity, dose ranging, route of administration, and therapeutic efficacy in non-human primates is a necessary next step in the clinical development of applying AAV gene therapy to treat patients with RA or AIDs.

## Materials and methods

### Antibodies and reagents

Antibodies specific to GFP (catalog no. 2555), HSP90 (catalog no. 4877), glyceraldehyde 3-phosphate dehydrogenase (GAPDH; catalog no. 5174), P-ERK1/2 (catalog no. 4376), P-p38 (catalog no. 9211), P-JNK1/2 (catalog no. 4668), and P-NF-κB/p65 (catalog no. 3033) were purchased from Cell Signaling Technology. Recombinant TNF (catalog no. 210-TA), recombinant IL-1β/IL-1F2 (catalog no. 401-ML), and human IL-1Ra/IL-1F3 Quantikine ELISA kit (catalog no. DRA00B) were purchased from R&D Systems.

### Plasmid construction and rAAV9 production

The inflammation-inducible AAV plasmid (pAAV.*pIR-Egfp*) was generated by replacing the CBA promoter and intron in the pAAVsc-*CB6-Egfp* plasmid with the endogenous mouse *Id1* promoter (−1,075/–993) containing two SBEs; YY1-, EGR1-, and CREB/ATF-binding sites; and one BRE.^39^ Additionally, the *PB2* promoter containing two NF-κB-binding sites and one FosP-binding site^40^ was introduced along with the MBL intron (Figure S1A). The *Egfp* gene in both pAAVsc-*CB6-Egfp* and pAAV.*pIR-Egfp* plasmids was replaced with mCherry, gLuc, or secreted codon-optimized human IL-1Ra (*IL1RN*) (NM_173841) cDNA. All constructs were verified by sequencing before packaging into the rAAV9 capsid (Figure 1C). EGFP-expressing rAAV9 was used as a Vec. rAAV9 particles were produced by transient plasmid transfection in HEK293 cells, followed by purification via CsCl sedimentation. The purified viral particles were quantified using droplet-digital PCR (ddPCR) on a QX200 ddPCR system (Bio-Rad) with *Egfp* or *mCherry*-specific primer/probe sets. The DNA sequences are listed in Table S1.

### Animals

SKG mice were obtained from Dr. Shimon Sakaguchi (Kyoto University, Japan) and maintained on a BALB/c background. Mice were housed under controlled conditions, with up to five mice per cage, an ambient temperature of 21°C ± 2°C, circulating air, and constant humidity of 50% ± 10% in a 12-h light/dark cycle. They were provided with *ad libitum* access to a standard chow diet and monitored every 3 days for their food and water intake and signs of distress. Mice exhibiting severe distress, including general malaise, severe cachexia, or more than 20% body weight loss, were humanely euthanized in accordance with veterinary guidance. Euthanasia was performed using a carbon dioxide chamber, followed by cervical dislocation. Mouse genotypes were determined by PCR analysis of tail genomic DNA; primer sequences are available upon request. All animal procedures complied with the *NIH Guide for the Care and Use of Laboratory Animals* and were approved by the University of Massachusetts Chan Medical School Institutional Animal Care and Use Committee (protocol no. A-202200036).

### Mouse models of inflammatory arthritis

To induce inflammatory arthritis, 10-week-old female SKG mice were injected i.p. with curdlan (6 mg/kg, dissolved in PBS; Fujifilm Wako Pure Chemical, catalog no. 032-09902). Clinical peripheral joint inflammation scoring and ankle thickness measurement using a digital caliper were performed twice weekly according to an established protocol.^73^^,^^74^ Histological inflammation and articular erosions were assessed in paraffin-embedded hindlimb tissue sections. Hematoxylin and eosin (H&E)-stained sections were scored for inflammation, while H&E and adjacent TRAP-stained sections were used to score articular erosions following Standardized Microscopic Arthritis Scoring of Histological Sections (SMASH) recommendations for a standardized processing and microscopic scoring.^75^ Trabecular bone mass and cortical thickness in the femur, as well as bone erosions in the foot, were assessed using microcomputed tomography (microCT) and radiographic analyses. To evaluate the effects of gene therapy, a single dose of 2.5 × 10^13^ vg/kg rAAV9s carrying *Egfp* or sIL-1Ra was administered i.v. to 10-week-old female SKG mice 2 weeks prior to curdlan injection. We have previously demonstrated that 2.5 × 10^13^ vg/kg of i.v.-injected rAAV9 is most effective for the transduction of bone-resident cells, including osteoclasts, osteoblasts, and osteocytes, in mice under normal and inflammatory conditions.^45^^,^^46^^,^^76^

For the K/BxN STA model, KRN T cell transgenic mice^50^ were crossed with NOD mice. Arthritogenic serum was collected from arthritic progeny and 150 μL of the K/BxN serum was transferred to 8-week-old female WT mice (C57BL/6) via i.p. injection on days 0 and 2. Clinical peripheral joint inflammation scoring and ankle thickness measurements were performed every other day, as previously described.^74^ Histologic sample preparation and scoring of inflammation and articular erosions followed the same protocol as in the SKG model.^74^ A single dose of 2.5 × 10^13^ vg/kg of rAAV9 containing *pCBA-mCherry*, *pCBA-Egfp*, *pCBA-sIL-1Ra*, or *pIR-sIL-1Ra* was administered i.v. to 8-week-old female WT mice 1 week prior to K/BxN serum injection.

Given that IL-1Ra deletion in mice differentially impact body weights, fat, and bone growth depending on genetic background,^55^ *Il1rn*^−/−^ mice, purchased from The Jackson Laboratory (B6.129S7-*Il1r1*^*tm1Imx*^/J, catalog no. 003245), were further backcrossed with C57BL/6J mice (catalog no. 000664, The Jackson Laboratory), and maintained on a C57BL/6J background. Interestingly, our *Il1rn*^−/−^ mice on a C57BL/6 background exhibited growth retardation and severe bone loss, which results from abnormal growth plate structure and increased osteoclast activity. These phenotypes are consistent with those observed in mice with constitutively active NLRP3 inflammasome signaling.^57^ DIRA is known to cause constitutive activation of the NLRP3 inflammasome, a mechanism implicated in NOMID.^11^ *Il1rn*^−/−^ mice were allowed to develop inflammatory arthritis until 15 weeks of age. Histological samples from femurs and ankles were prepared and scored as described above. Trabecular bone mass and cortical thickness in the femur, along with bone erosions in the foot, were assessed using microCT and radiographic analyses. To evaluate early intervention, a single dose of 2.5 × 10^13^ vg/kg of rAAV9 carrying pCBA.EGFP, pCBA.sIL-1Ra or pIR.sIL-1Ra was administered i.v. to 3-week-old female *Il1rn*^−/−^ mice and their littermate controls.

### Complete blood cell count

Peripheral blood was collected from AAV-treated mice via cheek bleeding and immediately transferred into a microtainer EDTA tube. Samples were analyzed within 1 h at room temperature using an automated hematology analyzer (VetScan HM5, Zoetis). Complete blood cell count tests evaluated WBCs, RBCs, lymphocytes, monocytes, hemoglobin, and PLTs.

### ELISA assay for cytokine measurement

Peripheral blood was collected via cheek bleeding and processed for cytokine analysis. Multiplex analysis was conducted using the Luminex 200 system (Luminex) by Eve Technologies. A total of 32 inflammation-associated cytokines and chemokines (eotaxin, G-CSF, GM-CSF, IFN-γ, IL-1α, IL-1β, IL-2, IL-3, IL-4, IL-5, IL-6, IL-7, IL-9, IL-10, IL-12p40, IL-12p70, IL-13, IL-15, IL-17A, KC, LIF, IFN-γ-induced protein 10, LPS-induced CXC chemokine, monocyte chemoattractant protein-1, M-CSF, monokine induced by IFN-γ, macrophage inflammatory protein-1α [MIP-1α], MIP-1β, VEGF-A, TNF-α, MIP-2, and RANTES [regulated on activation, normal T cell expressed and secreted]) were simultaneously measured using Eve Technologies’ Mouse Cytokine 32-Plex Discovery Assay (MilliporeSigma) according to the manufacturer’s protocol. The assay sensitivity for these markers ranged from 0.3 to 30.6 pg/mL.

### Measurement of nAbs

Eight-week-old WT mice were injected i.v. with PBS, Vec, rAAV9.pCBA.IL-1Ra, or rAAV9.pIR.IL-1Ra. Cheek bleeding was performed 4 weeks after rAAV injection to collect serum. To measure serum levels of rAAV9 nAbs in rAAV-treated mice, sera were added into cells in the presence of rAAV9 carrying reporter gene (β-Gal) at different dilutions (5-, 10-, and ≥20-fold). The presence of anti-rAAV9 nAbs in the serum inhibits the AAV infection *in vitro*. Based on the inhibition of reporter gene expression, we quantitated anti-rAAV9 nAb levels in the serum.^77^

To measure serum levels of nAbs against human sIL-1Ra in pCBA.IL-1Ra-treated mice, HEK293 cells were transiently transfected with the NF-κB-responsive reporter gene (*PB2-Luc*) and *Renilla* reporter gene, and 24 h later, they were treated with PBS, rhIL-1Ra (catalog no. 280-PA, R&D Systems), or sera for 1 h in the absence or presence of human IL-1β. rhIL-1Ra, 30 ng/mL, corresponds to the amount detected in serum IL-1Ra from pCBA.IL-1Ra-treated mice. We performed a luciferase assay 12 h later to measure firefly Luc activity and then, normalized it to *Renilla*. The presence of anti-human sIL-1Ra nAbs in the serum diminishes the ability of secreted IL-1Ra to block IL-1β-induced NF-κB activation *in vitro*. Based on the reporter gene expression, we quantitated relative amounts of anti-human sIL-1Ra nAb in the serum.

### Flow cytometry analysis for immune cells

Multicolor flow cytometric analysis was performed using an FACSymphony A5 (BD Biosciences) with exclusion of 7-AAD^+^ cells and doublets. Data analysis was conducted with FlowJo software (version 10.10, BD Biosciences). Splenocytes were harvested from the spleens of AAV-treated *Il-1rn*^−/−^ and littermate controls, passed through a 40-μm cell strainer, and washed with cold fluorescence-activated cell sorting (FACS) buffer (0.5% BSA [fraction V] and 1 mM EDTA in pH 7.2 PBS). T and B lymphocytes were isolated using CD45/B220-conjugated or CD4-conjugated anti-mouse magnetic particles (IMag, BD Biosciences, catalog nos. 551513 and 551539). Myeloid cells were identified using the following fluorochrome-labeled antibodies: BV510-conjugated CD11b (M1/70, BioLegend, catalog no. 101263), fluorescein isothiocyanate (FITC)-conjugated LY6C (HKL4, BioLegend, catalog no. 128006), phycoerythrin (PE)-Cy7-conjugated LY6G (1A8, BioLegend, catalog no. 127617), PE-Cy7-conjugated B220 (RA3-6B2, BioLegend, catalog no. 103222), allophycocyanin-conjugated CD3ε (145-2C11, BD Pharmingen, catalog no. 553066), PerCP-Cy5.5-conjugated CD4 (GK1.5, Tonbo Biosciences, catalog no. 65-0041-4025), FITC-conjugated CD8 (53-6.7, Tonbo Biosciences, catalog no. 35-0081-4025). Prior to staining, cell suspensions were incubated in Fc block (2.4G2 hybridoma; American Type Culture Collection) to prevent nonspecific binding.

### MicroCT and radiography

MicroCT 35 scanner (Scanco Medical) was used for qualitative and quantitative assessment of trabecular and cortical bone microarchitecture. The investigator conducting the analysis was blinded to the genotypes and treatment of the animals. Femurs and ankles were excised and scanned at spatial resolutions of 7 and 12 μm, respectively. Trabecular bone analysis of the distal femur was performed by contouring an upper 2.1-mm region, beginning 280 μm proximal to the growth plate. Cortical bone analysis of femur and tibia was conducted at the midshaft region (0.6) and three-dimensional (3D) reconstruction images were generated using distance transformation-based methods applied to binarized two-dimensional images. Alternatively, the Inveon multimodality 3D visualization program (Siemens Medical Solutions) was used for 3D rendering of fused static and dynamic microCT modalities. All images presented are representative of the respective genotypes (*n* > 5).

Whole-body radiographic images were obtained using the Trident Specimen Radiography System (Hologic) after euthanasia. The X-ray beam intensity was set to 1 mA 28–30 kV with automatic exposure control for consistent image acquisition (*n* > 5).

### Biomechanical analysis

Femora were mechanically tested in three-point bending using an electrical force mechanical testing machine (Electroforce 3230, Bose Corporation) at the Center for Skeletal Research Imaging and Biomechanical Testing Core (NIH, P30AR066261). The bending fixture had a bottom span length of 8 mm. The test was performed with the load point in displacement control moving at a rate of 0.05 mm/s, with force and displacement data collected at 60 Hz. All of the bones were consistently oriented during testing, with the cranial surface resting on the supports and being loaded in tension. Bending rigidity (EI, N ⋅ mm^2^), apparent modulus of elasticity (Eapp, MPa), ultimate moment (Mult, N-mm), apparent ultimate stress (σapp, MPa), work to fracture (Wfrac, mJ), and apparent toughness (Uapp, mJ/mm^3^) were calculated based on the force and displacement data from the tests and the mid-shaft geometry measured with microCT. Work to fracture is the energy required to cause femoral fracture, and it was calculated by finding the area under the force-displacement curve using the Riemann sum method. Bending rigidity was calculated using the linear portion of the force-displacement curve. The minimum MOI (Imin) was used when calculating the apparent modulus of elasticity.

### Histology and immunohistochemistry

For histology, femurs and ankles were dissected from the mice, fixed in 10% neutral buffered formalin for 2 days, and decalcified by 15% tetrasodium EDTA for 2–3 weeks. Tissues were dehydrated through a series of ethanol washes, washed twice in xylene, infiltrated with paraffin twice, and embedded in paraffin. The femur was sectioned at 6-μm thickness along the coronal plane from anterior to posterior, while ankle was sectioned following the SMASH-recommended sagittal sectioning plane of a hind paw, which is a more talus-oriented approach to assess four to five joints for evaluation. Sections were stained with H&E, toluidine blue, or TRAP as previously described.^46^

### Osteoblast and osteoclast culture and differentiation

Primary COBs were isolated from the calvaria of WT neonates (C57BL/6J) at postnatal day 3 using collagenase type II (50 mg/mL; Worthington, catalog no. LS004176)/dispase II (100 mg/mL; Roche, catalog no. 10165859001) and cultured in α-minimum essential medium (MEM) medium (Corning) containing 10% fetal bovine serum (FBS; Corning), 2 mM l-glutamine (Corning), 1% penicillin/streptomycin (Corning), and 1% nonessential amino acids (Corning). Cells were transduced with rAAV9 carrying pCBA.EGFP or pIR.EGFP, and 3 days later, they were stimulated with various inflammatory cytokines for 1 day. For osteogenic differentiation, cells were maintained in α-MEM containing 10% FBS, 2 mM l-glutamine, 1% penicillin/streptomycin, and 1% nonessential amino acids and differentiated with ascorbic acid (200 μM; Sigma, catalog no. A8960) and β-glycerophosphate (10 mM; Sigma, catalog no. G9422). GFP expression was assessed by immunoblotting with an anti-GFP antibody or by fluorescence microscopy.

For preparation of BMMs, femurs were dissected from 8-week-old WT (*Il1rn*^*+/+*^) and KO (*Il1rn*^−/−^) mice. Cells were collected by flushing and then plated overnight in α-MEM with 10% FBS. Non-adherent cells were collected and cultured in the presence of M-CSF (20 ng/mL; R&D Systems, catalog no. 416-ML-010) for 24 h to obtain monocytes. Cells were transduced with rAAV9 carrying pCBA.EGFP, pIR.EGFP, Vec, pCBA.IL-1Ra, or pIR.IL-1Ra, then differentiated into osteoclasts 1 day later in the presence of M-CSF (20 ng/mL) and RANKL (10 ng/mL; R&D Systems, catalog no. 462-TEC-010). Cells were stimulated with inflammatory cytokines for 1 day. GFP expression was assessed by immunoblotting with an anti-GFP antibody or by fluorescence microscopy. Alternatively, multinucleated osteoclasts were stained with TRAP, and the expression of osteoclastic genes was measured by RT-PCR.

### Luciferase assay

The inflammation-responsive reporter gene (pAAV.*pIR-gLuc*) and *Renilla* gene (Promega) were transfected into HEK293 cells using the Effectene transfection reagent (Qiagen). At 24 h later, the cells were stimulated with human TNF (10 ng/mL), IL-1β/IL-1F2 (20 ng/mL), or BMP4 (50 ng/mL, R&D Systems, catalog nos. 210-TA, 201-LB, and 314-BP). The NF-κB-responsive reporter gene (*PB2-Luc*) and *Renilla* gene were transfected into HEK293 cells along with various doses of human IL-1Ra plasmid, and 24 h later, the cells were stimulated with human IL-1β/IL-1F2. Alternatively, HEK293 cells were transfected with the NF-κB-responsive reporter gene (*PB2-Luc*) and *Renilla* gene, and 24 h later, the cells were treated with human IL-1β/IL-1F2 and various amounts of the supernatant harvested from human IL-1Ra-expressing HEK293 cells. After 24 h of stimulation, dual luciferase assays were performed according to the manufacturer’s protocol (Promega), and luciferase activity was normalized to *Renilla.*

### Quantitative RT-PCR analysis

Total RNA was isolated from cells using QIAzol (Qiagen) and cDNA was synthesized using the High-Capacity cDNA Reverse Transcription Kit from Applied Biosystems. Quantitative RT-PCR was performed using SYBR Green PCR Master Mix (Bio-Rad) with the CFX connect RT-PCR detection system (Bio-Rad). Tissues were snap-frozen in liquid nitrogen for 30 s and homogenized in 1 mL QIAzol for 1 min to measure mRNA levels of human *IL1RN*, *GFP*, *mCherry*, *mouse Il-1rn*, *Ila*, *Il1b*, *Il6*, *Cstk*, *Acp5*, and *Ctr* in various tissues and cells. Primers used for PCR are shown in Table S1.

### RNA-seq and whole-transcriptome analysis

Synovial tissues were harvested from AAV-treated SKG and STA mice, and total RNA was isolated using QIAzol (Qiagen), followed by column-based purification with the Purelink RNA Mini Kit (Invitrogen, catalog no. 12183018A). RNA integrity was assessed using the RNA integrity number (RIN) or RNA quality number (RQN), which were measured with an Agilent Bioanalyzer 2100. Samples with RIN/RQN values greater than 9.0 were selected for sequencing and processed by Innomics. RNA-seq was performed using the DNBSEQ platform, with a paired-end read length of 150 bp (PE150). Bioinformatics analysis was conducted by Via Scientific, using DESeq2 for DE analysis. For quality control, FASTQC (version 0.11.8) was used to assess raw read quality. To remove unwanted sequences, Bowtie2 or STAR was used to filter out common rRNA and tRNA reads. RNA-seq reads were then aligned to the mouse genome (mm10) using HISTAT2. Gene and isoform expression levels were quantified using RSEM with reference transcriptomes. DEBrowser (version 1.29.3) was used for exploratory analysis and interactive visualization, provided by Via Scientific. DE analysis was performed with DESeq2_1.28.1.^78^ Within DE analysis, ‘ashr’ was used to create log2 fold change (LFC) shrinkage for each comparison. Significant DEGs were filtered with the criteria false discovery rate less than 0.05 and absolute |LFC| >0.585. GSEA was performed with GSEA.^79^

### Statistical analysis and reproducibility

All experiments were performed with *n* ≥ 3 biological replicates and repeated independently two or three times to ensure reproducibility. This applies to immunofluorescence, immunohistochemistry, histology, immunoblotting, and RT-PCR. Data are presented as mean ± standard deviation (SD). All statistical analyses were performed using GraphPad Prism (version 10.4.0). To assess normality, the Shapiro-Wilk test was performed and a quantile-quantile plot was visually inspected for additional confirmation of normality. If the Shapiro-Wilk test yielded *p* > 0.05, indicating no significant deviation from normality, then a two-tailed, unpaired Student’s t test was used for comparisons between two groups. If *p* ≤ 0.05, indicating deviation from normality, then a Mann-Whitney *U* test was applied instead. For comparisons involving three or more groups, a one-way ANOVA was conducted if the Shapiro-Wilk test suggested normality. The Brown-Forsythe test was used to assess homogeneity of variances. If variances were found to be unequal (*p* ≤ 0.05), then a Welch’s ANOVA was performed instead. All ANOVA tests were followed by Dunnett’s T3 post hoc test for pairwise comparisons. Statistical significance was defined as *p* < 0.05.

## Data availability

Data supporting the findings of this manuscript are available from the corresponding authors upon request. The raw data are protected and are not available due to data privacy laws. The minimum dataset generated in this study necessary to interpret, verify, and extend the research in the article is provided in the supplemental information.

## Acknowledgments

We thank Thomas Gallagher for reviewing this manuscript and the many individuals who provided valuable reagents. We also thank the UMass Chan Flow Cytometry Core and the Imaging and Biomechanical Testing Core of the Center for Musculoskeletal Research funded by 10.13039/100000002NIH core instrument grants (NIH S10OD028576 and NIH P30AR075042). G.G. is supported by grants from the NIH (R01NS076991, P01HL131471, R01AI121135, UG3HL147367, R01HL097088, R01HL152723, U19AI149646, and UH3HL147367). J.-H.S. is supported by grants from the NIH/National Institute of Arthritis and Musculoskeletal and Skin Diseases (R01AR086572, R01AR078230, and R21AR084644), UMass Center for Clinical and Trnaslational Science Pilot Project Program, the Li Weibo Institute for Rare Diseases Research Pilot Grant Program, and Dong-A ST.

## Author contributions

Y.-S.Y. designed, executed, and interpreted the experiments. J.X. and H.M. designed and generated all of the AAVs used in this work. S.C., M.-J.K., N.D., S.L., and E.M. analyzed the immune cells, bone marrow cells, and skeletal phenotypes. K.-Y.L. and E.G. reviewed the manuscript. G.G. and J.-H.S. supervised the research and prepared the manuscript.

## Declaration of interests

G.G. and J.-H.S. have submitted a patent application for the methodology described in this study. G.G. and J.-H.S. are scientific co-founders of AAVAA Therapeutics and hold equity in the company. G.G. is also a scientific co-founder of Voyager Therapeutics, Adrenas Therapeutics, and Aspa Therapeutics and holds equity in these companies. G.G. is an inventor on patents with potential royalties licensed to Voyager Therapeutics, Adrenas Therapeutics, Aspa Therapeutics, and other biopharmaceutical companies.

## Supplemental information

Document S1. Figures S1–S9 and Tables S1 and S2

Document S2. Article plus supplemental information
