# Supplementary material for: Nature-inspired IL-1 targeted therapy to treat chronic inflammatory diseases
Source: Mol Ther. 2025 Sep 10;33(12):6379–97. doi: 10.1016/j.ymthe.2025.09.008 (PMC12466080; doi:10.1016/j.ymthe.2025.09.008)
Supplement: Document S1. Figures S1–S9 and Tables S1 and S2 [file mmc1.pdf]

YMTHE, Volume *33*

Supplemental Information

Nature-inspired IL-1 targeted therapy to treat chronic inflammatory diseases

Yeon-Suk Yang, Mi-Jeong Kim, Sachin Chaugule, Emma Mayer, Ngoc DeSouza, Hong Ma, Jun Xie, Ki-Young Lee, Shaoguang Li, Ellen Gravallese, Guangping Gao, and Jae-Hyuck Shim

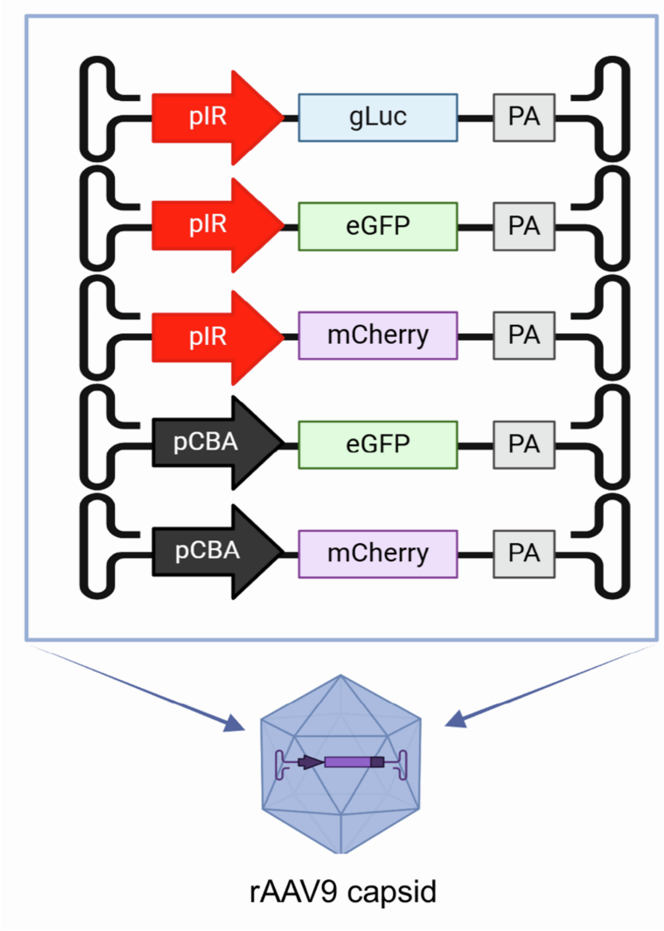

Figure S1. Schematic diagram of the AAV constructs encoding reporter genes.

Gaussian luciferase (gLuc), enhanced green fluorescent protein (eGFP), and mCherry were cloned into the AAV plasmids containing the CBA promoter (constitutive expression) or the IR promoter (inflammation-inducible expression). The plasmids were packaged into rAAV. pCBA: CMV enhancer/chicken β-actin promoter, PA: polyadenylation. Created with biorender.com.

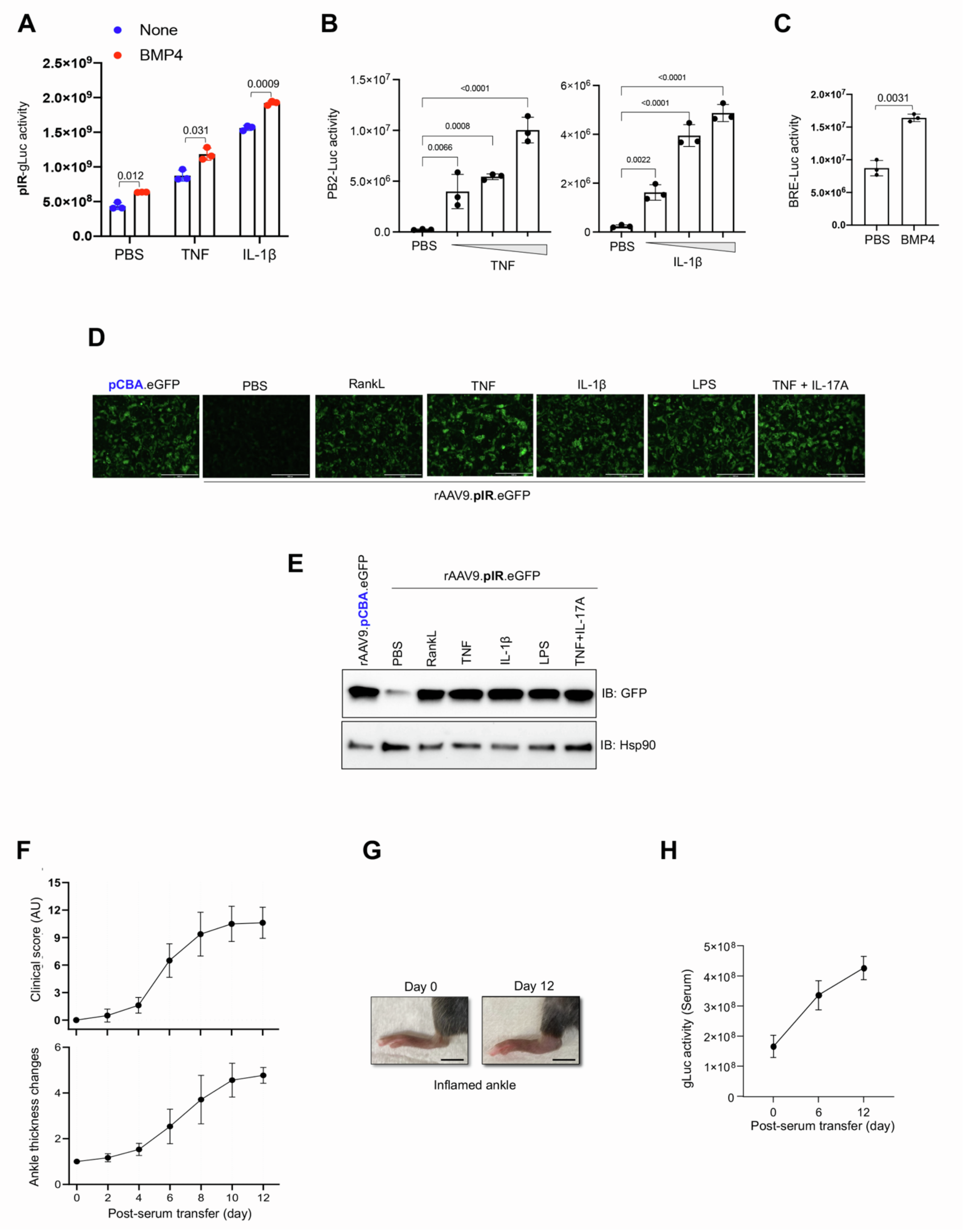
A
None
BMP4
pIR-gLuc activity
2.5×10^9
2.0×10^9
1.5×10^9
1.0×10^9
5.0×10^8
0.0
0.012
0.031
0.0009
PBS
TNF
IL-1β
B
PB2-Luc activity
1.5×10^7
1.0×10^7
5.0×10^6
0.0
<0.0001
0.0008
0.0066
PBS
TNF
6×10^6
4×10^6
2×10^6
0
<0.0001
<0.0001
0.0022
PBS
IL-1β
C
BRE-Luc activity
2.0×10^7
1.5×10^7
1.0×10^7
5.0×10^6
0.0
0.0031
PBS
BMP4
D
pCBA.eGFP
PBS
RankL
TNF
IL-1β
LPS
TNF + IL-17A
rAAV9.pIR.eGFP
E
rAAV9.pCBA.eGFP
rAAV9.pIR.eGFP
PBS
RankL
TNF
IL-1β
LPS
TNF+IL-17A
IB: GFP
IB: Hsp90
F
Clinical score (AU)
15
12
9
6
3
0
Ankle thickness changes
6
4
2
0
0
2
4
6
8
10
12
Post-serum transfer (day)
G
Day 0
Day 12
Inflamed ankle
H
gLuc activity (Serum)
5×10^8
4×10^8
3×10^8
2×10^8
1×10^8
0
0
6
12
Post-serum transfer (day)

Figure S2. ***In vitro and in vivo*** **characterization of inflammation-inducible rAAV vector.**

A. HEK293 cells were transiently transfected with the pIR-gLuc plasmid and 24 hours later, stimulated with PBS, TNF, or IL-1β in the absence or presence of BMP4. 12 hours later, a luciferase assay was performed to measure gLuc activity (n = 3). **B.** HEK293 cells were transiently transfected with the NF-κB-responsive reporter gene (*PB2-Luc*) and *Renilla* reporter gene and 24 hours later, stimulated with different concentrations of TNF or IL-1β. 12 hours later, a luciferase assay was performed to measure firefly Luc activity and then, normalized to *Renilla* (n = 3). PBS was used as a negative control. **C.** HEK293 cells were transiently transfected with the BMP-responsive reporter gene (*BRE-Luc*) and *Renilla* reporter gene and 24 hours later, stimulated with PBS or BMP4 (100 ng/ml). 12 hours later, a luciferase assay was performed to measure firefly Luc activity and then, normalized to *Renilla* (n = 3). **D, E.** Wild-type bone marrow-derived monocytes (BMMs) were transduced with rAAV9 carrying pCBA.eGFP or pIR.eGFP and cultured under osteoclast differentiation conditions in the presence of PBS or various inflammatory cytokines. 6 days later, GFP expression was assessed by fluorescence microscopy **(D)** and by immunoblotting with an anti-GFP antibody **(E)**. Anti-HSP90 antibody was used as a loading control. PBS was used as a negative control. Scale bar: 100 μm. **F-H.** 2-month-old wild-type mice were injected i.p. with arthritic K/BxN serum (days 0 and 2) 7 days after i.v. injection of rAAV9.pIR.gLuc (- day 7) and euthanized (day 12). Ankle thickness and clinical joint inflammation were measured every other day (n = 4, **F**). Photographic images showing inflamed ankle joints at two different time points (**G**). Scale bars: 3 mm. Harvested blood was subjected to a luciferase assay (n = 4, **H**). Data are representative of two independent experiments (**D, E, G**). Values represent mean ± SD and groups were compared using an unpaired two-tailed Student's *t*-test (**C**) or one-way ANOVA test (**A, B, F, H**).

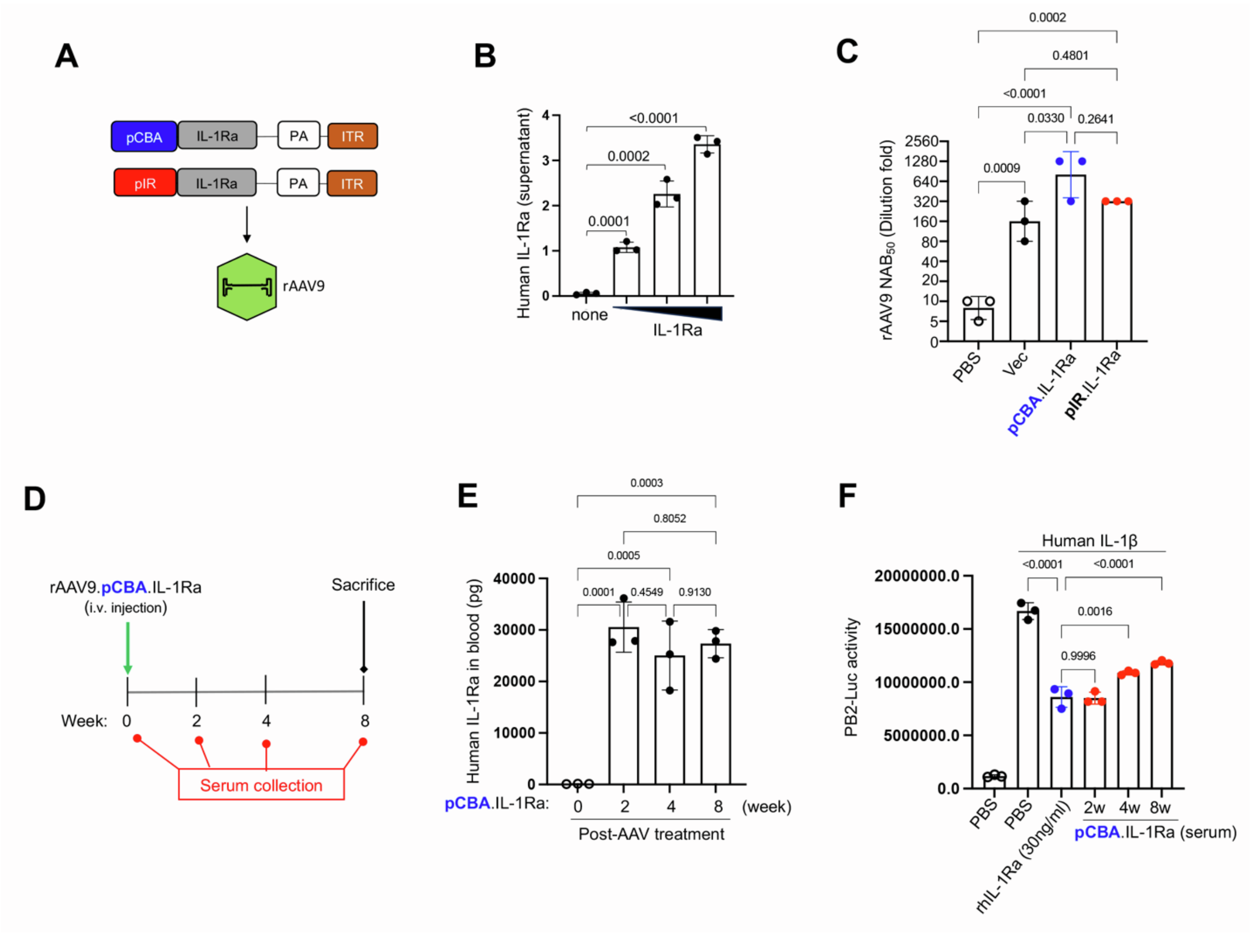

Figure S3. Characterization of human sIL-1Ra-expressing AAV vector.

A. Codon-optimized human secreted IL-1Ra (*IL1RN*) cDNA was cloned into the AAV plasmid with the CBA or IR promoter and then packaged into rAAV9 capsid. PA, polyadenylation; ITR, Inverted terminal repeat. **B.** HEK293 cells were transiently transfected with different concentrations of pCBA.IL-1Ra plasmid. 2 days later, the harvested supernatant was subjected to ELISA (n = 3). **C**. 8-week-old wild type mice were injected i.v. with PBS, vector control (Vec), rAAV9.pCBA.IL-1Ra, or rAAV9.pIR.IL-1Ra. Cheek bleeding was performed 4 weeks after rAAV injection and serum levels of neutralizing antibody against AAV9 capsids were assessed (n = 3). **D-F.** Diagram of the study and treatment methods **(D)**. 8-week-old wild type mice were injected i.v. with rAAV9.pCBA.IL-1Ra. Cheek bleeding was performed right before AAV

injection (0 week) and 2, 4, 8 weeks after the injection. Protein levels of human IL-1Ra in blood was measured by ELISA (n = 3, **E**). HEK293 cells were transiently transfected with the NF-κB-responsive reporter gene (*PB2-Luc*) and *Renilla* reporter gene and 24 hours later, treated with PBS, recombinant human IL-1Ra (30 ng/ml), or serum for 1 hour in the absence or presence of human IL-1β. 12 hours later, a luciferase assay was performed to measure firefly Luc activity and then, normalized to *Renilla* (n = 3, **F**). Values represent mean ± SD and groups were compared using one-way ANOVA test (**B, C, E, F**).

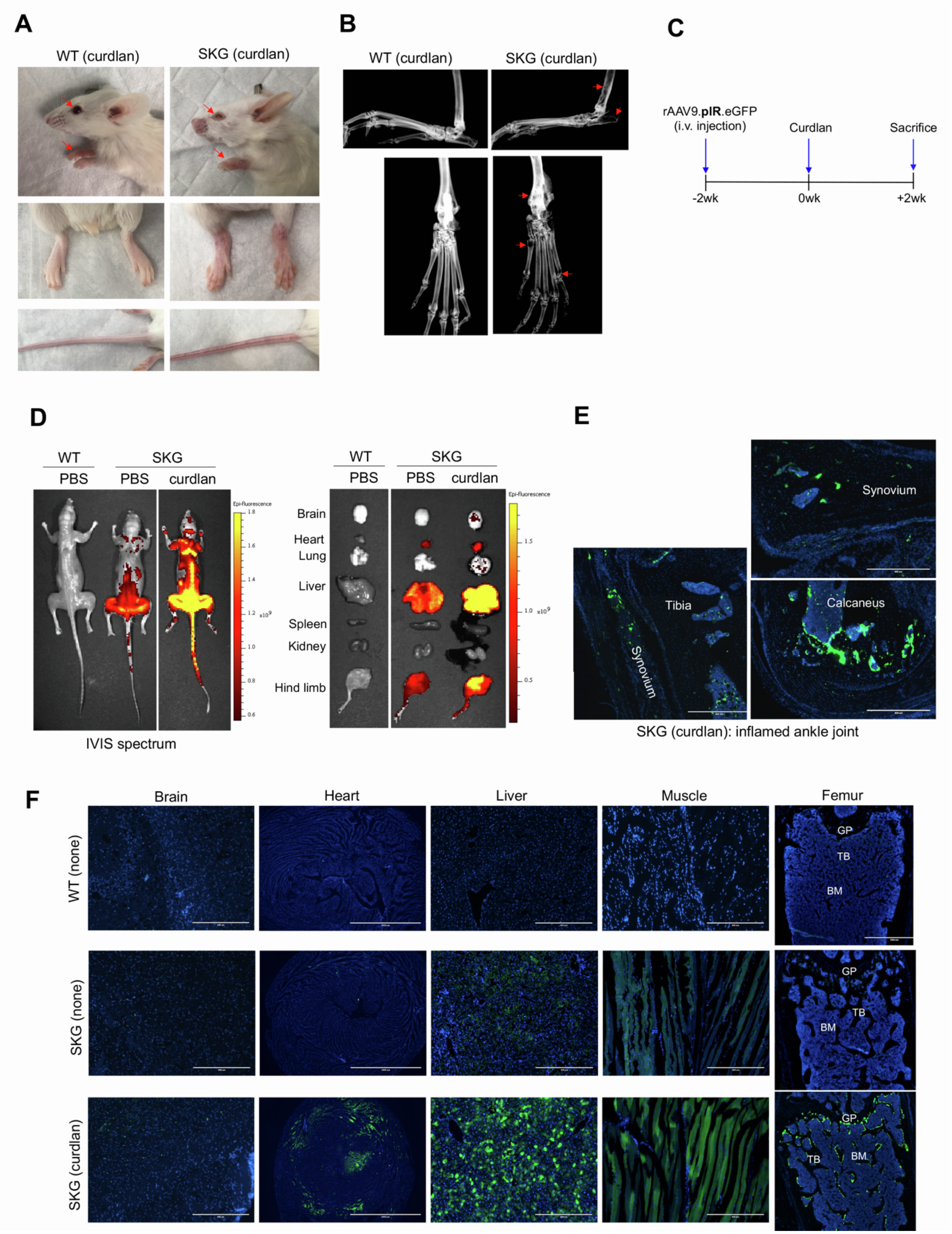
A
WT (curdlan)
SKG (curdlan)
B
WT (curdlan)
SKG (curdlan)
C
rAAV9.pIR.eGFP
(i.v. injection)
Curdlan
Sacrifice
-2wk
0wk
+2wk
D
WT
SKG
PBS
PBS
curdlan
IVIS spectrum
Brain
Heart
Lung
Liver
Spleen
Kidney
Hind limb
E
Synovium
Tibia
Calcaneus
SKG (curdlan): inflamed ankle joint
F
Brain
Heart
Liver
Muscle
Femur
WT (none)
SKG (none)
SKG (curdlan)
GP
TB
BM

Figure S4. Biodistribution and expression of inflammation-inducible rAAV9 in arthritic SKG mice.

A, B. 12-week-old WT and SKG mice were injected with curdlan. 6 weeks later, photographic and radiographic images show severe inflammation in eyes, hands, feet, joints, and tails in curdlan-treated SKG mice, but not in curdlan-treated wild-type mice. Red arrows indicate inflammation (**A**) and bone erosions (**B**). **C–F.** Diagram of the study and treatment methods. 12-week-old WT and SKG mice were injected i.p. with PBS or curdlan 2 weeks after i.v. injection of rAAV9.pIR.eGFP (**C**). 2 weeks later, GFP expression in whole body and individual tissues was monitored using an IVIS optical imaging system (**D**) and fluorescence microscopy on cryosectioned tissues (**E, F**). Inflamed ankle joints of curdlan-treated SKG mice show a subset of GFP-expressing cells in the synovium and calcaneus bones **(E)**. Scale bars: 400 μm (**E, F-left**), 1000 μm (**F-right**). GP: growth plate, TB: trabecular bone, BM: bone marrow. Representative images of four replicates are displayed (**A, B, D, E, F**).

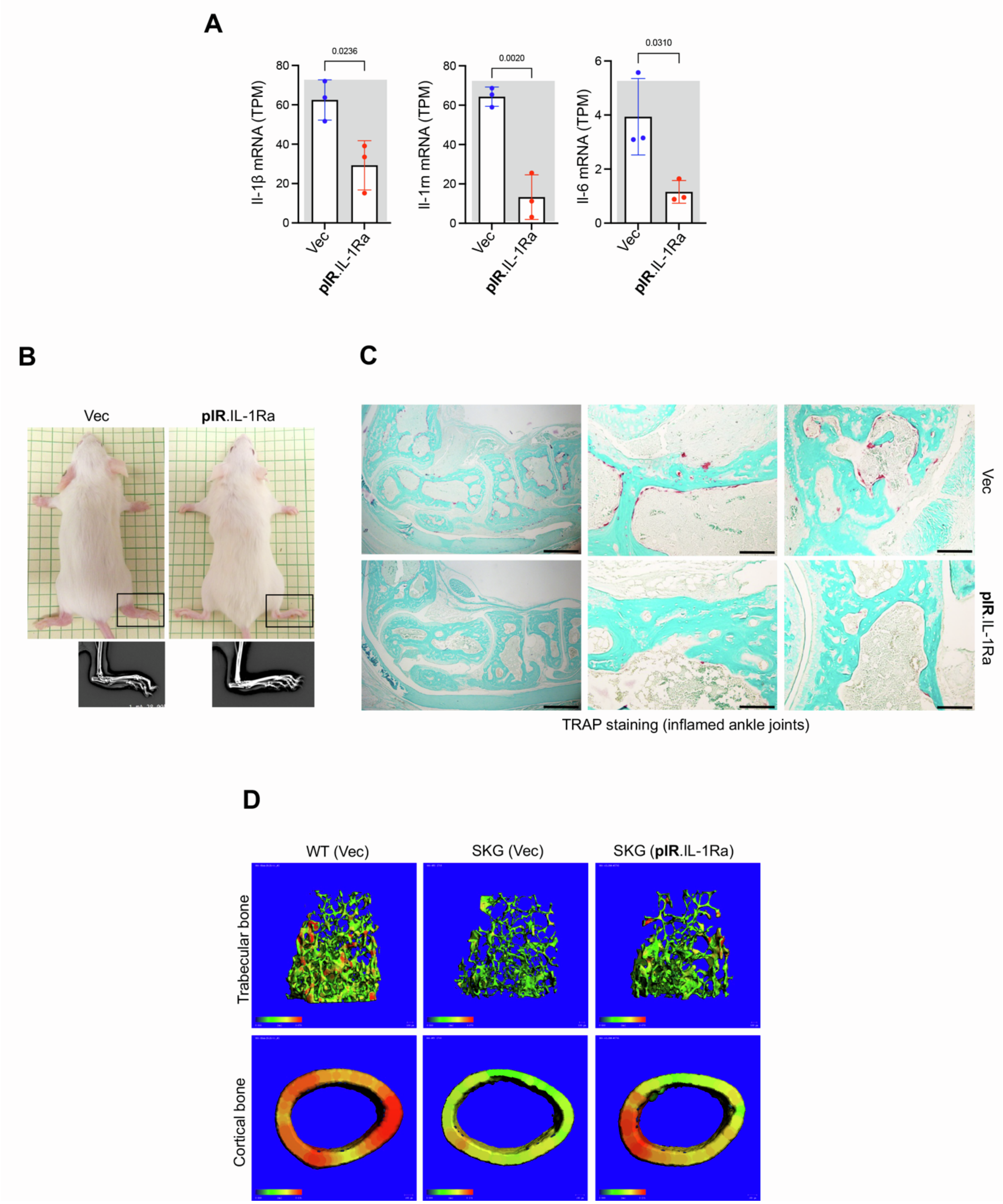
A
0.0236
Il-1β mRNA (TPM)
Vec
pIR.IL-1Ra
0.0020
Il-1m mRNA (TPM)
0.0310
Il-6 mRNA (TPM)
B
Vec
pIR.IL-1Ra
C
Vec
pIR.IL-1Ra
TRAP staining (inflamed ankle joints)
D
WT (Vec)
SKG (Vec)
SKG (pIR.IL-1Ra)
Trabecular bone
Cortical bone

Figure S5. Inflammation-inducible expression of human sIL-1Ra reduces IL-1 signaling, inflammation, and bone loss in arthritic SKG mice.

12-week-old WT and SKG mice were injected i.p. with curdlan 2 weeks after i.v. injection of rAAV9 carrying vector control (Vec) or pIR.IL-1Ra (n = 6). 6 weeks later, total RNA in synovial tissues was subjected to bulk RNA sequencing. **A.** A significant decrease in *Il1b, Il1rn*, and *Il6* transcripts in mice treated with pIR.IL-1Ra relative to control (n = 3). TPM, transcripts per million. Grey boxes indicate curdlan-treated SKG mice. **B.** Photographic and radiographic images showing inflamed ankle joints. **C.** TRAP-stained sections of ankle joints show a significant decrease in TRAP-positive osteoclasts in mice treated with pIR.IL-1Ra relative to Vec. Scale bar: 100 μm. **D.** MicroCT analysis shows 3D reconstitution of trabecular and cortical bones from AAV-treated WT and SKG femurs. Scale bar: 100 μm**.** A two-tailed unpaired Student's *t*-test was used for comparing two groups (**A**). Representative images of six replicates are displayed (**B-D**).

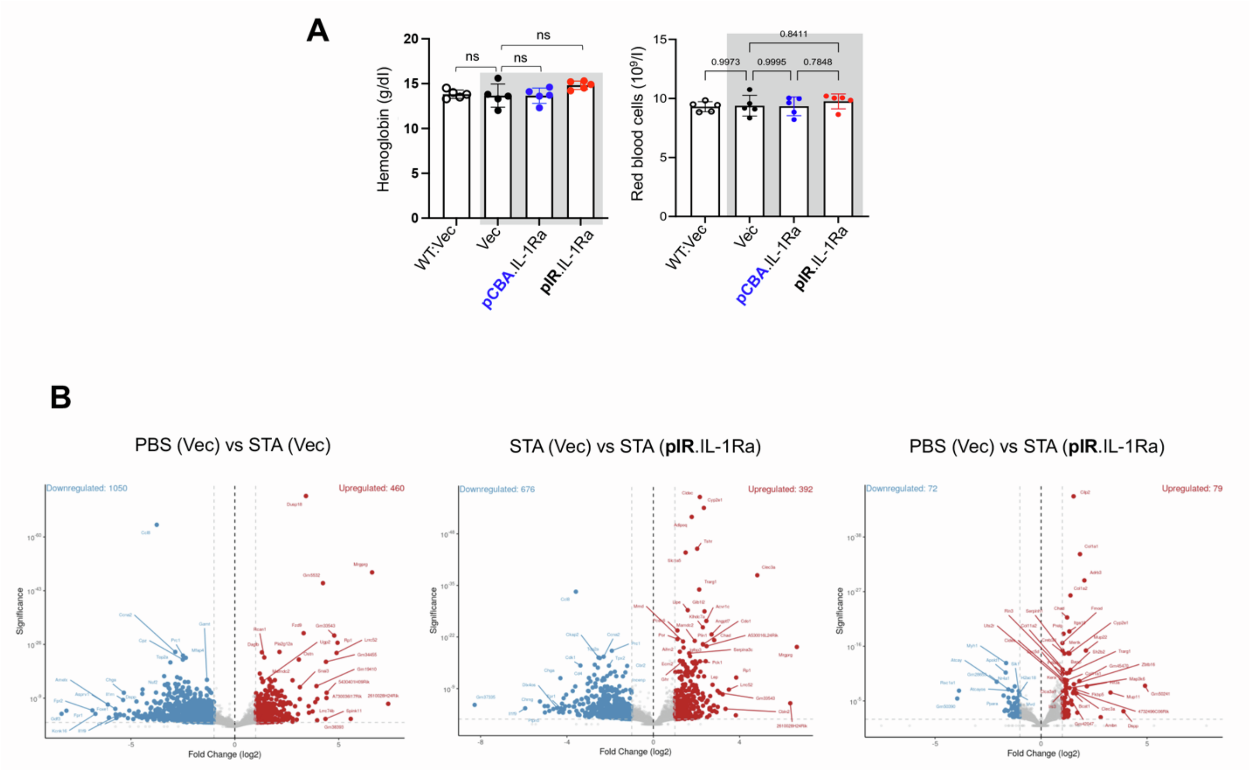

Figure S6. AAV-mediated expression of human sIL-1Ra alters gene expression in arthritic K/BxN STA mice.

2-month-old wild-type mice were injected i.p. with PBS or arthritic K/BxN serum (STA) 1 week after i.v. injection of rAAV9 carrying vector control (Vec), pCBA.IL-1Ra, or pIR.IL-1Ra (n = 5). **A.** 12 days later, a complete blood count test of peripheral blood was performed, showing no difference in hemoglobin and red blood cells in rAAV-treated mice. Grey boxes indicate K/BxN STA mice. **B.** Total RNA in synovial tissues obtained from the AAV-treated mice was subjected to bulk RNA sequencing (n = 3). A volcano plot showing differential gene expression is displayed. Values represent mean ± SD and groups were compared using a one-way ANOVA test (**A**).

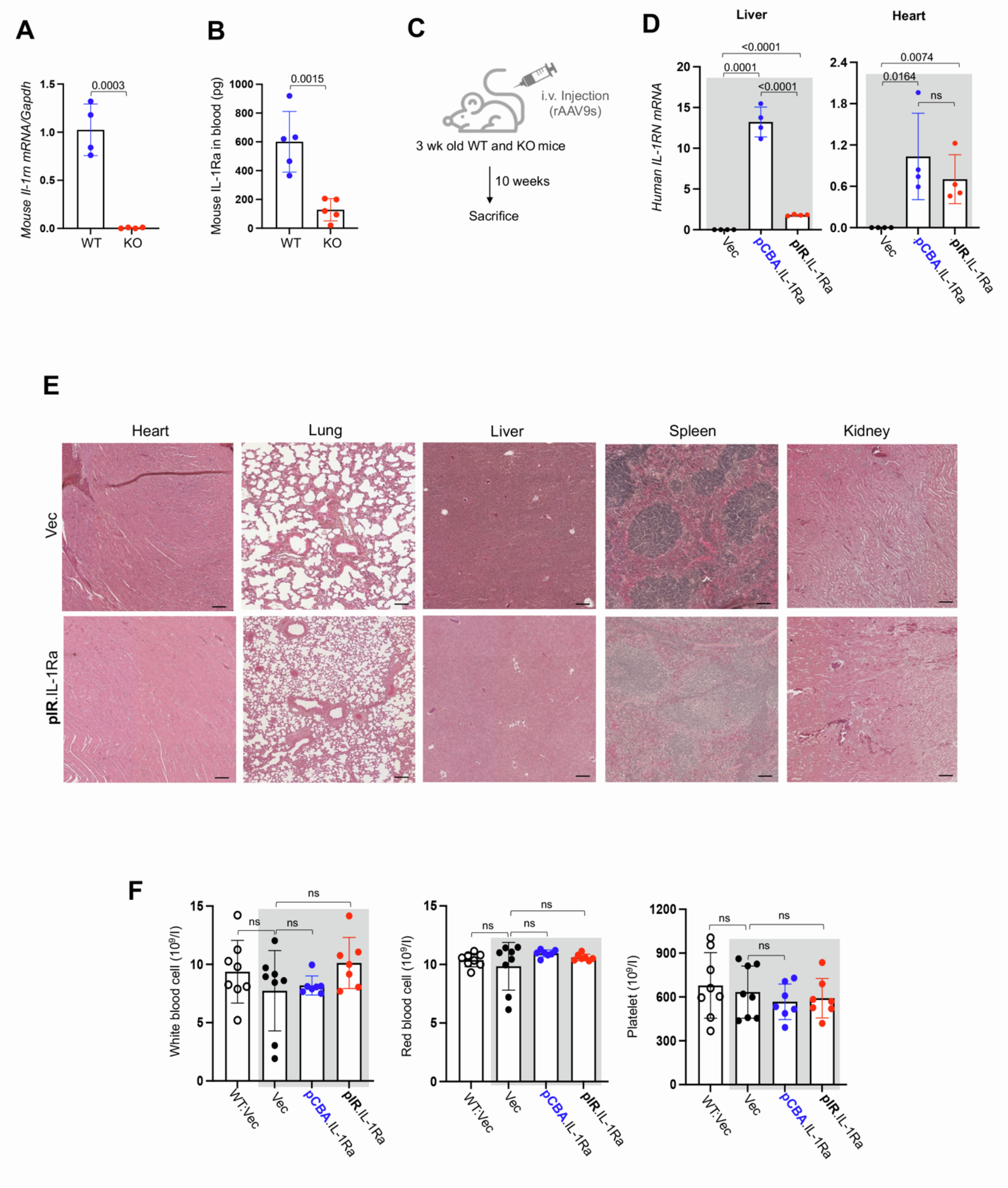
A
B
C
D
Mouse Il-1rn mRNA/Gapdh
0.0003
WT
KO
Mouse IL-1Ra in blood (pg)
0.0015
i.v. Injection
(rAAV9s)
3 wk old WT and KO mice
10 weeks
Sacrifice
Liver
Heart
Human IL-1RN mRNA
<0.0001
0.0001
<0.0001
0.0074
0.0164
ns
Vec
pCBA.IL-1Ra
pIR.IL-1Ra
E
Heart
Lung
Liver
Spleen
Kidney
Vec
pIR.IL-1Ra
F
White blood cell (10⁹/l)
Red blood cell (10⁹/l)
Platelet (10⁹/l)
ns
WT:Vec
Vec
pCBA.IL-1Ra
pIR.IL-1Ra

Figure S7. Effects of sIL-1Ra-expressing rAAV9 in DIRA mice.

A, B. mRNA levels of mouse *Il1rn* in the liver (n = 4, **A**) and protein levels of mouse IL-1Ra in the peripheral blood (n = 5, **B**) of WT ($Il1rn^{+/+}$) and KO ($Il1rn^{-/-}$) mice were measured by qPCR and ELISA, respectively. **C-F.** Diagram of the study and treatment methods. 3-week-old WT and KO mice were injected i.v. with rAAV9 carrying vector control (Vec), pCBA.IL-1Ra, or pIR.IL-1Ra. **(C)**. 10 weeks later, mRNA levels of human sIL-1Ra in liver and heart were measured by qPCR (n = 4, **D**). H&E staining of the longitudinal sections of the AAV-treated tissues were performed, demonstrating no grossly apparent effects on heart, lung, liver, spleen, and kidney (n= 3, **E**). Complete blood count test of peripheral blood was performed, demonstrating no difference in white blood cells, red blood cells, and platelet in AAV-treated mice (n = 8, **F**). Grey boxes indicate KO mice. Values represent mean ± SD and groups were compared using a two-tailed unpaired Student's *t*-test for comparing two groups (**A, B**) or one-way ANOVA test (**D, F**).

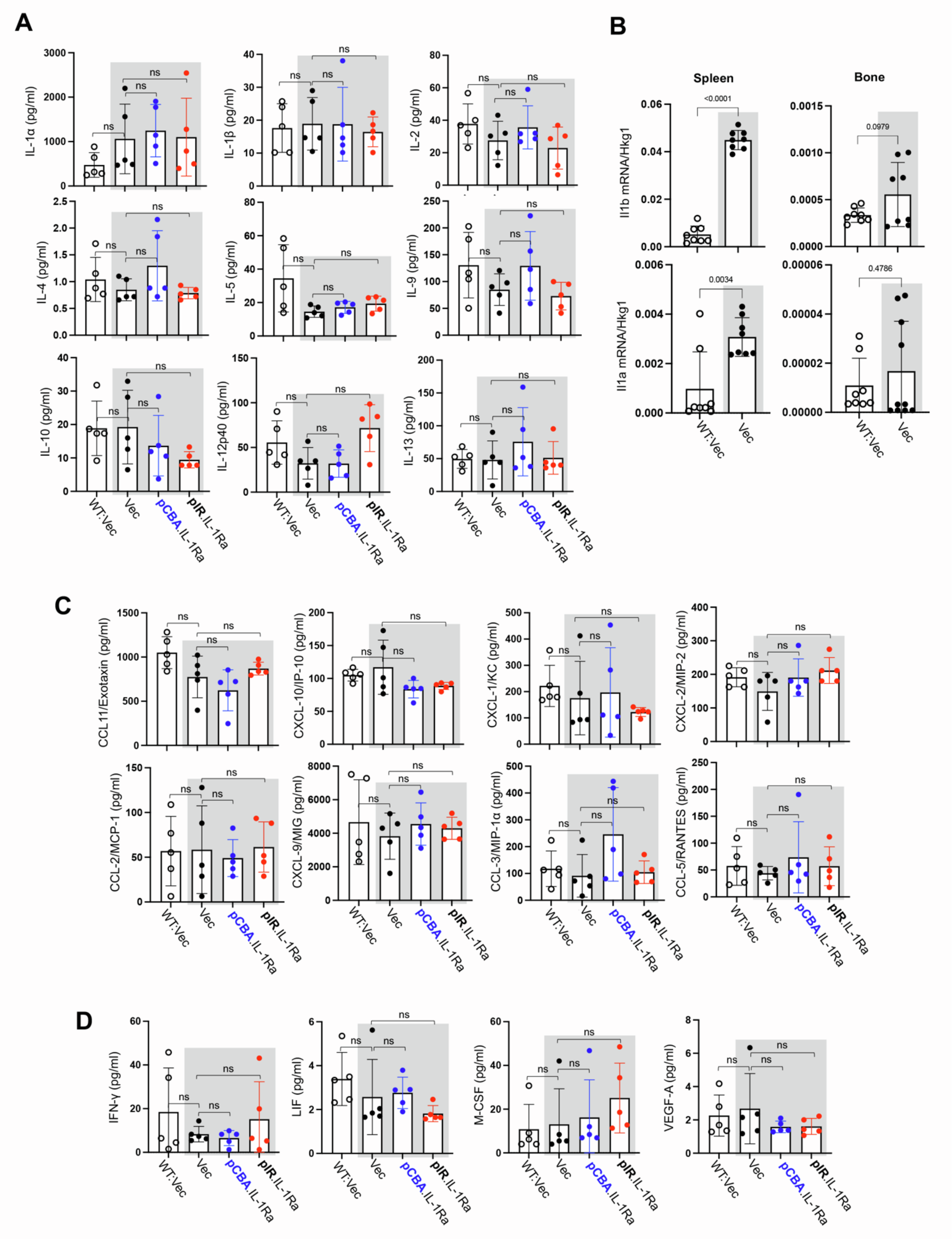
A
IL-1α (pg/ml)
IL-1β (pg/ml)
IL-2 (pg/ml)
IL-4 (pg/ml)
IL-5 (pg/ml)
IL-9 (pg/ml)
IL-10 (pg/ml)
IL-12p40 (pg/ml)
IL-13 (pg/ml)
ns
WT:Vec
Vec
pCBA.IL-1Ra
pIR.IL-1Ra
B
Spleen
Bone
Il1b mRNA/Hkg1
Il1a mRNA/Hkg1
<0.0001
0.0979
0.0034
0.4786
C
CCL11/Exotaxin (pg/ml)
CXCL-10/IP-10 (pg/ml)
CXCL-1/KC (pg/ml)
CXCL-2/MIP-2 (pg/ml)
CCL-2/MCP-1 (pg/ml)
CXCL-9/MIG (pg/ml)
CCL-3/MIP-1α (pg/ml)
CCL-5/RANTES (pg/ml)
D
IFN-γ (pg/ml)
LIF (pg/ml)
M-CSF (pg/ml)
VEGF-A (pg/ml)

Figure S8. Effects of sIL-1Ra-expressing rAAV9 on inflammatory cytokines and chemokines in DIRA mice.

3-week-old WT (*Il1rn*$^{+/+}$) and KO (*Il1rn*$^{-/-}$) were injected i.v. with rAAV9 carrying vector control (Vec), pCBA.IL-1Ra, or pIR.IL-1Ra. 10 weeks later, protein levels of interleukins (**A**), chemokines (**C**), IFN-γ, LIF, M-CSF, and VEGF-A (**D**) in the peripheral blood of rAAV-treated mice were measured by ELISA (n = 5). mRNA levels of *Il1a* and *Il1b* in the spleen and tibia of AAV-treated WT and KO mice were measured by qPCR (n = 8, **B**). Grey boxes indicate KO mice. Values represent mean ± SD and groups were compared using a one-way ANOVA test.

A

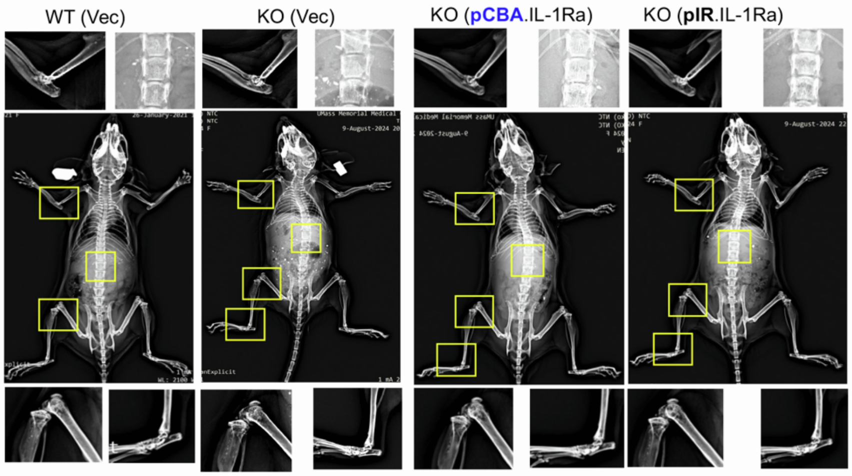
WT (Vec)
KO (Vec)
KO (pCBA.IL-1Ra)
KO (pIR.IL-1Ra)

B

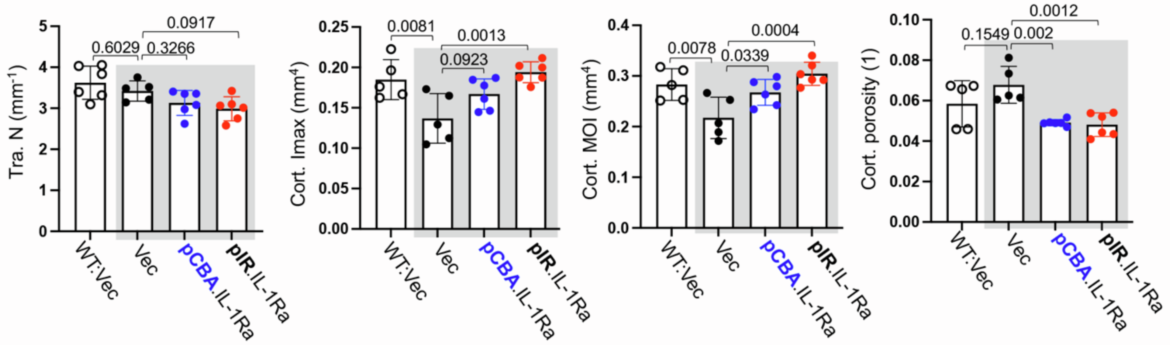
Tra. N (mm-1)
0.6029
0.3266
0.0917
Cort. Imax (mm4)
0.0081
0.0013
0.0923
Cort. MOI (mm4)
0.0078
0.0339
0.0004
Cort. porosity (1)
0.1549
0.002
0.0012
WT:Vec
Vec
pCBA.IL-1Ra
pIR.IL-1Ra

C

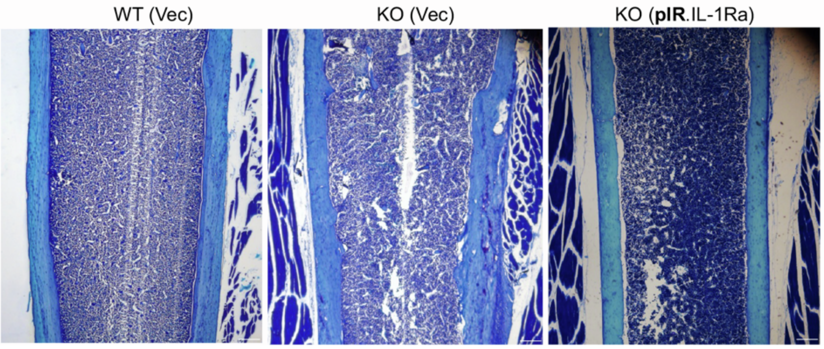
WT (Vec)
KO (Vec)
KO (pIR.IL-1Ra)

Figure S9. AAV-mediated expression of human sIL-1Ra ameliorates skeletal phenotypes in DIRA mice. 3-week-old WT (*Il1rn*$^{+/+}$) and KO (*Il1rn*$^{-/-}$) mice were injected i.v. with rAAV9 carrying vector control (Vec), pCBA.IL-1Ra, or pIR.IL-1Ra. **A.** 10 weeks later, radiographic analysis of a whole body was performed. Compared to Vec-treated WT mice, Vec-treated KO mice show small body size and short stature, which was reversed by treatment with pCBA.IL-1Ra or pIR.IL-1Ra (n = 5). **B.** microCT analysis shows the quantification of trabecular bone number (Tra. N) and cortical bone moment of inertia (MOI), maximum moment of inertia (Imax), and porosity in rAAV-treated femurs. Grey boxes indicate KO mice. **C.** Low magnification of toluidine blue–stained femoral sections of AAV-treated mice. The same experiment was performed in **Figure 5C**. Scale bar: 1 mm. Values represent mean ± SD and groups were compared using a one-way ANOVA test.

Movie S1: 3-week-old WT (*Il1rn*$^{+/+}$) and KO (*Il1rn*$^{-/-}$) mice were injected i.v. with rAAV9 carrying vector control (Vec) or pIR.IL-1Ra and mouse behaviors were assessed 10 weeks post-injection (n = 5). Movie shows the body size and mobility of 13-week-old AAV-treated mice, including a Vec-treated WT mouse (normal body size, mobility, and activity level), a Vec-treated KO mouse (reduced body size, mobility, and activity level), and a pIR.IL-1Ra-treated KO mouse (normal body size, mobility, and activity level). The arrow indicates the pIR.IL-1Ra-treated KO mouse.

Table S1: DNA sequences of AAV plasmids

Inflammation-inducible promoter	GCTTCGCGCCCTAAGTCTGCAGGTGACGGGCTCAGGGGCGGGGGCTGGGTGGGGGG<br>GAGCGGAGAATGCTCCAGCCCAGTTTGCCGTCTCCATGGCGACCGCCCGCGCGGCG<br>CCAGCCTGACAGCCCGTCCGGGTTTTATGAATGGGTGACGTCACGGGCCTGGCGTCT<br>AACGGTCTGAGCCGCTTGTTCAGACGCTGACACAGACCAGCCCGGGAAAGGTGAGC<br>TCACAGAGGGGACTTTCCGAGAGATCTACAGAGGGGACTTTCCGAGAGCGAGCTTG<br>GGCTGCAGGTCGACCGTCCATCCATTCACAGCGCTTCTATAAAGGCGCCAGCTGAGG<br>CGCCTACTACTCCAACCGCGACTGCAGCGAGCAACTGAGAAGACTGGATAGAGCCG<br>GCGGTTCCGCGAACGAGCAGTGACCGCGCTCCCACCCAGCTCTGCTCTGCAGCTCCA<br>CCAGTGTCTCTCTAGA
MBL intron	TCAGATCGCCTGGAGACGCCATCCACGCTGTTTTGACCTCCATAGAAGACACCGGGA<br>CCGATCCAGCCTCCGCGGCCGGGAACGGTGCATTGGAACGCGGATTCCCCGTGCCA<br>AGAGTGACGTAAGTACCGCCTATAGAGTCTATAGGCCCACCCCCTTGGCTTCTTATG<br>CATGCTATACTGTTTTTGGCTTGGGGTCTATACACCCCCGCTTCCTCATGTTTGCTGC<br>CCGTGACCAGCACGTCAACGATTTTGTGGGCACGGGCGACACCGCAGTGTAGTCTG<br>AGCAGTACTCGTTGCTGCCGCGCGCGCCACCAGACATAATAGCTGACAGACTAACA<br>GACTGTTCCTTTCCATGGGTCTTTTCTGCA
CBA promoter	TCTCCCCATCTCCCCCCCCTCCCCACCCCCAATTTTGTATTTATTTATTTTTTAATTAT<br>TTTGTGCAGCGATGGGGGCGGGGGGGGGGGGGGGGGGCGCGCGCCAGGCGGGG<br>CGGGGCGGGGCGAGGGGCGGGGCGGGGCGAGGCGGAGAGGTGCGGCGGCAGCCAA<br>TCAGAGCGGCGCGCTCCGAAAGTTTCCTTTTATGGCGAGGCGGCGGCGGCGGC<br>CCTATAAAAAGCGAAGCGCGCGGCGGGCGGGAGCGGGATC
CBA intron	GTGAGCGGGCGGGACGGCCCTTCTCCTCCGGGCTGTAATTAGCGCTTGGTTTAATGA<br>CGGCTTGTTTCTTTTCTGTGGCTGCGTGAAAGCCTTGAGGGGCTCCGGGAGGGCCCT<br>TTGTGCGGGGGGAGCGGCTCGGGGGGTGCGTGCGTGTGTGTGTGCGTGGGGAGCGC<br>CGCGTGCGGCTCCGCGCTGCCCGGCGGCTGTGAGCGCTGCGGGCGCGGCGCGGGGC<br>TTTGTGCGCTCCGCAGTGTGCGCGAGGGGAGCGCGGCCGGGGGCGGTGCCCCGCGG<br>TGCGGGGGGGGCTGCGAGGGGAACAAAGGCTGCGTGCGGGGTGTGTGCGTGGGGG<br>GGTGAGCAGGGGGTGTGGGCGCGTCGGTCGGGCTGCAACCCCCCCTGCACCCCCCT<br>CCCCGAGTTGCTGAGCACGGCCCGGCTTCGGGTGCGGGGCTCCGTACGGGGCGTGG<br>CGCGGGGCTCGCCGTGCCGGGCGGGGGGTGGCGGCAGGTGGGGGTGCCGGGCGGG<br>GCGGGGCCGCCTCGGGCCGGGGAGGGCTCGGGGGAGGGGCGCGGCGGCCCCCGGA<br>GCGCCGGCGGCTGTCGAGGCGCGGCGAGCCGCAGCCATTGCCTTTTATGGTAATCGT<br>GCGAGAGGGCGCAGGGACTTCCTTTGTCCCAAATCTGTGCGGAGCCGAAATCTGGG<br>AGGCGCCGCCGCACCCCCTCTAGCGGGCGCGGGGCGAAGCGGTGCGGCGCCGGCAG<br>GAAGGAAATGGGCGGGGAGGGCCTTCGTGCGTCGCCGCGCCGCCGTCCCCTTCTCC<br>CTCTCCAGCCTCGGGGCTGTCCGCGGGGGGACGGCTGCCTTCGGGGGGGACGGGGC<br>AGGGCGGGGTTCGGCTTCTGGCGTGTGACCGGCGGCTCTAGAGCCTCTGCTAACCAT<br>GTTCATGCCTTCTTCTTTTTCCTACAGCTCCTGGGCAACGTGCTGGTTATTGTGCTGT<br>CTCATCATTTTGGCAAAG
Codon-optimized human sIL-1Ra	ATGGAAATCTGCAGAGGCCTCCGCAGTCACCTAATCACTCTCCTCCTCTTCCTGTTCC<br>ATTCAGAGACGATCTGCCGACCCTCTGGGAGAAAATCCAGCAAGATGCAAGCCTTC<br>AGAATCTGGGATGTTAACCAGAAGACCTTCTATCTGAGGAACAACCAACTAGTTGCT<br>GGATACTTGCAAGGACCAAATGTCAATTTAGAAGAAAAGATAGATGTGGTACCCAT<br>TGAGCCTCATGCTCTGTTCTTGGGAATCCATGGAGGGAAGATGTGCCTGTCCTGTGT<br>CAAGTCTGGTGATGAGACCAGACTCCAGCTGGAGGCAGTTAACATCACTGACCTGA<br>GCGAGAACAGAAAGCAGGACAAGCGCTTCGCCTTCATCCGCTCAGACAGTGGCCCC<br>ACCACCAGTTTTGAGTCTGCCGCCTGCCCCGGTTGGTTCCTCTGCACAGCGATGGAA<br>GCTGACCAGCCCGTCAGCCTCACCAATATGCCTGACGAAGGCGTCATGGTCACCAA<br>ATTCTACTTCCAGGAGGACGAGTAGTAA

Table S2: Sequences of RT-PCR primers

Gene	Forward	Reverse
Human *IL-1RN*	GCCTCCGCAGTCACCTAATC	TGACACAGGACAGGCACATC
Mouse *Il-1rn*	TGTGCCTGTCTTGTGCCAAGTC	GCCTTTCTCAGAGCGGATGAAG
Mouse *Il1a*	ACGGCTGAGTTTCAGTGAGACC	CACTCTGGTAGGTGTAAGGTGC
Mouse *Il1b*	TGGACCTTCCAGGATGAGGACA	GTTCATCTCGGAGCCTGTAGTG
mCherry	GAACGGCCACGAGTTCGAGA	CTTGGAGCCGTACATGAACTGAGG
Egfp	AGCAAAGACCCCAACGAGAA	GGCGGCGGTCACGAA
Mouse *Ctsk*	AGCAGAACGGAGGCATTGACTC	CCCTCTGCATTTAGCTGCCTTTG
Mouse *Acp5*	GCGACCATTGTTAGCCACATACG	CGTTGATGTCGCACAGAGGGAT
Mouse *Ctr*	CTTCCATGCTGATCTTCTGG	CAGATCTCCATTGGGCACAA
Mouse *Gapdh*	ACTGAGCAAGAGAGGCCCTA	TATGGGGGTCTGGGATGGAA
Mouse *Rplp0*	TGGCCAATAAGGTGCCAGCTGCTG	CTTGTCTCCAGTCTTTATCAGCTGCAC
